# The effects of graded levels of calorie restriction: I. impact of short term calorie and protein restriction on body composition in the C57BL/6 mouse

**DOI:** 10.18632/oncotarget.4142

**Published:** 2015-06-06

**Authors:** Sharon E. Mitchell, Zhanhui Tang, Celine Kerbois, Camille Delville, Penelope Konstantopedos, Aurélie Bruel, Davina Derous, Cara Green, Richard M. Aspden, Simon R. Goodyear, Luonan Chen, Jackie J.D. Han, Yingchun Wang, Daniel E.L. Promislow, David Lusseau, Alex Douglas, John R. Speakman

**Affiliations:** ^1^ Institute of Biological and Environmental Sciences, University of Aberdeen, Aberdeen, Scotland, UK; ^2^ Institute of Medical Sciences, University of Aberdeen, Aberdeen, Scotland, UK; ^3^ Key laboratory of Systems Biology, Shanghai Institute of Biological Sciences, Chinese Academy of Sciences, Shanghai, China; ^4^ Chinese Academy of Sciences Key Laboratory of Computational Biology, Chinese Academy of Sciences, Max Planck Partner Institute for Computational Biology, Shanghai Institutes for Biological Sciences, Chinese Academy of Sciences, Shanghai, China; ^5^ State Key Laboratory of Molecular Developmental Biology, Institute of Genetics and Developmental Biology, Chinese Academy of Sciences, Chaoyang, Beijing, China; ^6^ Department of Pathology and Department of Biology, University of Washington, Seattle, WA, USA

**Keywords:** food intake, dietary restriction, protein restriction, calorie restriction, body composition

## Abstract

Faced with reduced levels of food, animals must adjust to the consequences of the shortfall in energy. We explored how C57BL/6 mice withdrew energy from different body tissues during three months of food restriction at graded levels up to 40% (calorie restriction: CR). We compared this to the response to equivalent levels of protein restriction (PR) without a shortfall in calories. Under CR there was a dynamic change in body mass over 30 days and thereafter it stabilized. The time to reach stability was independent of the level of restriction. At the end of three months whole body dissections revealed differential utilization of the different tissues. Adipose tissue depots were the most significantly utilized tissue, and provided 55.8 to 60.9% of the total released energy. In comparison, reductions in the sizes of structural tissues contributed between 29.8 and 38.7% of the energy. The balance was made up by relatively small changes in the vital organs. The components of the alimentary tract grew slightly under restriction, particularly the stomach, and this was associated with a parallel increase in assimilation efficiency of the food (averaging 1.73%). None of the changes under CR were recapitulated by equivalent levels of PR.

## INTRODUCTION

The impact of reducing the level of food intake (variously called dietary restriction or calorie restriction CR) [1, 2] on animal lifespan was discovered almost 100 years ago [3]. Since that time there has been an expanding interest in the generality of the effect across different species [4, 5], and the impact of different levels of CR on healthspan and lifespan [6, 7]. There has also been much recent interest in the potentially important roles of different macronutrients within the diet [8] and the importance of background genotype on the magnitude and direction of the effect [9-14]. In addition there has been a large effort for at least the last 3 decades to try and discover the underlying molecular mechanisms by which CR exerts its effects [6, 15-20].

When an animal is first placed onto CR it faces an immediate discrepancy between the number of calories it is ingesting and the number of calories it is expending. The only way to bridge this immediate gap is to withdraw energy that is stored in its tissues. All tissues contain energy that can be metabolized to make good the shortfall. They vary however in their energy density, and hence utility as an energy supply. Fat tissue, contains more calories per gram than lean body tissue, and therefore might be considered the ideal source of energy to meet the gap between intake and demand. Indeed up until relatively recently many considered that this was the primary function of adipose tissue: to provide an energy reserve to meet the immediate energy shortfall when supply fails. Following the discovery of leptin [21], a hormone produced by white adipocytes, it has become clear that adipose tissue also performs many vital endocrine roles [22]. As CR continues, and fat reserves are finite, there is a need to bring back into balance the level of expenditure with the level of intake. This could be achieved by increasing the level of energy extraction from the food [23] or by reducing energy expenditure. This latter response might be accomplished by reducing levels of physical activity, reducing body temperature, or by reducing the sizes of the organs in the body which utilize energy at the highest rates. Such organs for example include the brain, heart, kidneys, alimentary tract and liver [24]. Reducing the sizes of these tissues may, however, compromise their metabolic functions. Hence animals face a complex choice in which tissues they should utilize, and which they should preserve. Some tissues, however, may be readily sacrificed, such as the reproductive organs. Indeed, one of the ideas behind the lifespan increasing effects of CR is that when individuals shut down reproductive physiology and behavior, resources are instead diverted towards somatic maintenance [25, 26].

Animals responding to CR therefore face a complex set of decisions about which tissues they should withdraw energy from, so that they are able to meet the immediate shortfall and reduce their energy demands, yet maintain as much as possible metabolic functioning. To clarify, although we use the terms ‘choice’ and ‘decision’ with respect to these processes, following the terminology of many previous studies regarding energy allocation ‘decisions’, we do not mean to imply that these processes involve any conscious decision making on the part of the animals involved. Moreover, while this terminology also presumes that the changes observed are ‘adaptive’ it should always be borne in mind that this may not be the case. Although many studies have explored the impacts of CR on the sizes of individual tissues [27-29], very few studies have provided a comprehensive analysis of the responses across the entire body e.g. [30] and [31], and none previously at graded levels of restriction. In the current study we sought to make such a comprehensive body composition analysis. Since the need to modulate the sizes of the energy consuming tissues would be greater when the level of restriction is greater, it would be anticipated that the extent of response would in some way be linked to the extent of CR. How do animals change their decisions about what tissues to protect and which ones to utilize as the levels of restriction change? To address this issue we explored the patterns of change in relation to graded levels of CR from 0 to 40%. Finally, it has been suggested that the responses to reducing the availability of food depend less on the shortfall in calories, and more on the shortfall of specific macronutrients, notably protein [32]. We therefore contrasted the responses of the animals to graded levels of CR (where both calorie and protein supply change in tandem) to the responses to graded restrictions only in the levels of dietary protein (hereafter called protein restriction (PR)).

## RESULTS

### Calorie restriction (CR)

#### Food intake

**Figure 1:**
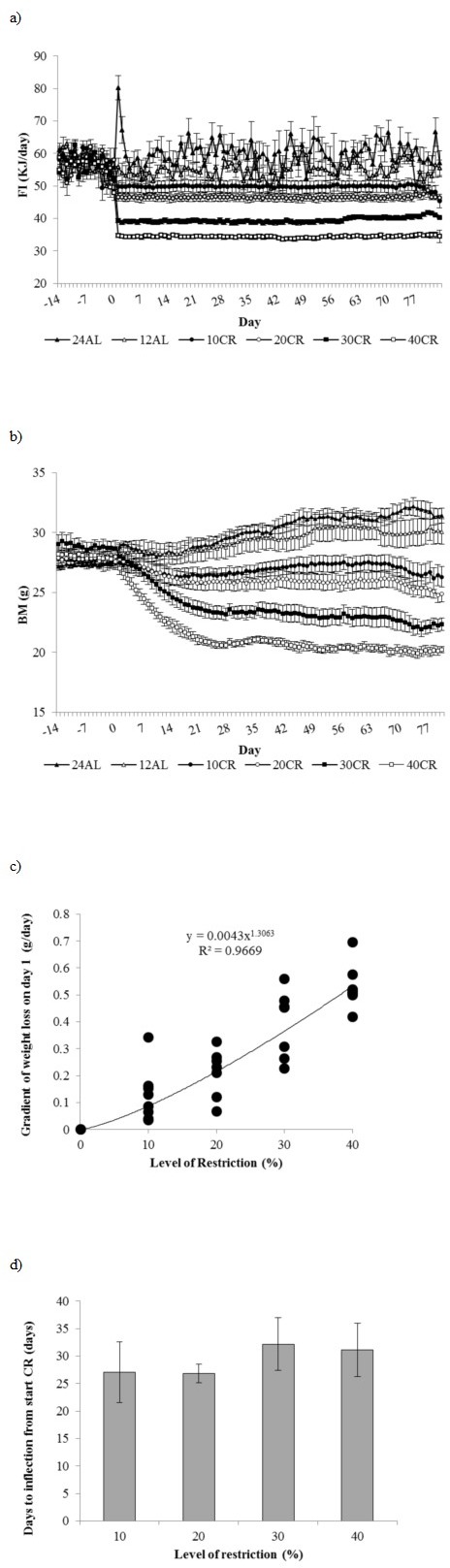
Food intake and body mass changes recorded over 3 months graded calorie restriction Daily records of **a.** food intake (FI) (kJ/day) and **b.** body mass (BM) (g) recorded over 2 weeks of baseline monitoring (days −14 to −1) and 12 weeks of treatment comprising 12 or 24h *ad libitum* (AL) feeding and graded levels of caloric restriction (CR) from 10 to 40% (10CR, 20CR, 30CR and 40CR respectively). Data are presented as daily mean ± SEM (g). **c.** Initial rates of weight loss for the four graded restriction groups. The value is the sum of the coefficients in x and x^2^ of the second order fitted polynomial to the body weights over the first 30 days of restriction. **d.**Time taken (days) for weight loss to slow to zero in relation to the extent of restriction. The inflection points were calculated from the fitted polynomial curves to the weight loss data of days 1 to 30 inclusive. Error bars are SEM.

All mice which were exposed to the same 12 hour *ad libitum* (12AL) feeding regime during baseline quickly acclimated to only having their food available during darkness and consumed their daily energy requirements within the restricted time that it was accessible (Figure 1a). The mean food intake recorded over the baseline period across all the mice was 56.09 ± 0.26 kJ/day (*n* = 49 individuals measured over 14 days) (Figure 1a). The protein intake over the same period averaged 0.668 grams per day. No significant difference between the six restricted groups was found over this period (One way ANOVA, F_(5, 48)_ = 1.26, *p* > 0.05). When the 24 hour *ad libitum* (24AL) group was first given 24h access to food there was a short period of about 3 days of hyperphagia (Figure 1a). Food intake increased by 44% on day 1 to 80.2 ± 3.64 kJ/day, but this then returned to baseline levels by day 4. Across the restriction phase, average food intake over the entire period was not significantly different between the two *ad libitum* groups and averaged 60.09 ± 1.71 kJ/day in the 24AL group and 54.81 ± 1.82 kJ/day in the 12AL group (GLM-RM, F_(1, 15)_ = 4.46, *p* = 0.052). Because the CR animals always ate their entire ration, when the average level of restriction was expressed relative to the baseline intakes, the 10%, 20%, 30% and 40% restricted (10, 20, 30 and 40CR) groups achieved exactly these percentage levels of restriction. However, if the level of intake at the final week of restriction was compared to the average intakes of the 12AL or 24AL groups during the same weeks the estimated values of the percentage levels of restriction were slightly different at 9, 18, 29 and 39%, relative to 12AL, and 17, 24, 35 and 44% relative to 24AL group, for nominally 10, 20, 30 and 40CR groups respectively.

#### Body mass

No significant difference in average body mass was observed between groups over the baseline period (One way ANOVA F_(5, 48)_ = 1.03, *p* > 0.05) (Figure 1b). Over the three month treatment body mass varied significantly over time (RM-GLM, F_(80, 3440)_ = 26.74, *p* < 0.001), between diet groups (F_(5, 43)_ = 28.84, *p* < 0.001), with a significant diet by day interaction (F_(400, 3440)_ = 11.61, *p* < 0.001). The hyperphagic feeding response observed in the 24AL mice at the start of treatment was mirrored by an increase in body mass over first few days (Figure 1b). The rise lasted 3 days and then body mass declined for a period of 7 days before subsequently rising again over the remainder of the measurement period. Over the three month treatment period body mass increased in both the 12AL and 24AL groups by on average 1.88 ± 0.59g and 3.91 ± 0.52g respectively (paired *t*-test, t_14_ = 4.59, *p* < 0.01 and t_14_ = 7.18, *p* < 0.001 compared to baseline body mass respectively) (Figure 1b). In contrast body mass of the mice under CR started to decline in all 4 groups immediately after the treatment started (Figure 1b). This decline was significant compared to baseline body mass after just 1 day in both 30 and 40CR groups (paired *t*-test, t_14_ = 5.97 and 8.41, *p* < 0.001 respectively), by day 2 in the 20CR (paired *t*-test, t_14_ = 4.45, *p* < 0.01) and day 4 in the 10CR animals (paired *t*-test, t_14_ = 2.52, *p* < 0.05). Compared to the 12AL controls, body mass was significantly lower from days 4 and 11 onwards in the 40 and 30CR groups (One Way ANOVA F_(5, 43)_ = 3.373 and 11.05, respectively, *p* < 0.05, post hoc Tukey *p* < 0.05 respectively). The weight loss of the 20 and 10CR groups were more gradual and only significantly lower than 12AL from 20 and 54 days onwards respectively (One Way ANOVA F_(5, 43)_ = 22.65 and 40.05, *p* for both < 0.001, post hoc Tukey *p* < 0.05).

The initial rate of mass loss was approximately linear and then slowed until around day 25, after which no further mass loss occurred and in most individuals there was a slight rise (Figure 1b). We fitted polynomial curves to the mass loss trajectories of the individual mice over days 1 to 30 of restriction and in all cases (except 1) the best fit curves were second order. (For one animal under 20% restriction there appeared to be no weight loss over the first 30 days and so a curve could not be fitted). Parameters of the fitted curves are in supplementary materials Table S1. The rate of initial mass loss on day 1, reflected by the term of the polynomial in x plus the term in x^2^ (i.e. the tangent to the fitted curve on day x = 1) was positively related to the extent of restriction (ANOVA F_(3, 27)_ = 28.16, *p* < 0.0005). The relationship, however, was not linear (Figure 1c) and best described by a power function (r^2^ = 0.96 compared to 0.74 for linear fit). The exponent of 1.3 indicated that increasing levels of restriction provoked more extreme rates of mass loss than anticipated from the level of restriction alone. We calculated the points of inflection of these fitted polynomial curves from the coefficient of the term in x divided by twice the coefficient of the term in x^2^ (equal to the differentiated polynomial solved for x = 0). These inflection points, reflecting the day when mass change fell to zero, averaged 29.3 days across all the restriction groups (*n* = 31, se = 2.09), and were independent of the extent of restriction (Figure 1d : ANOVA F_(3, 27)_ = 0.40, *p* > 0.05). Hence the time taken for mass loss to stabilize in response to the restriction was independent of the extent of restriction.

Compared to the 12AL control group, a significantly lower body mass was observed in all CR groups at the end of treatment (one way ANOVA F_(5, 43)_ = 34.12, *p* < 0.01; all Tukey pairwise comparisons to 12 AL significant at *p* < 0.01, −3.75 ± 0.83 g with 10CR, −4.98 ± 0.67 g with 20CR, −7.43 ± 0.45 g with 30CR and −9.73 ± 0.32 g, with 40CR. The asymptotic levels of body mass relative to the 12AL controls at the end of treatment were 12.40 ± 2.75%, 16.44 ± 2.2%, 24.57 ± 1.5% and 32.18 ± 1.07% in 10, 20, 30 and 40 CR groups, respectively. In contrast the 24AL group ended the experiment on average 0.85 g ± 0.61 g (2.81 ± 2.03%) heavier than the 12AL animals.

#### Dual energy x-ray absorptiometry (DXA) analysis of body composition

**Figure 2:**
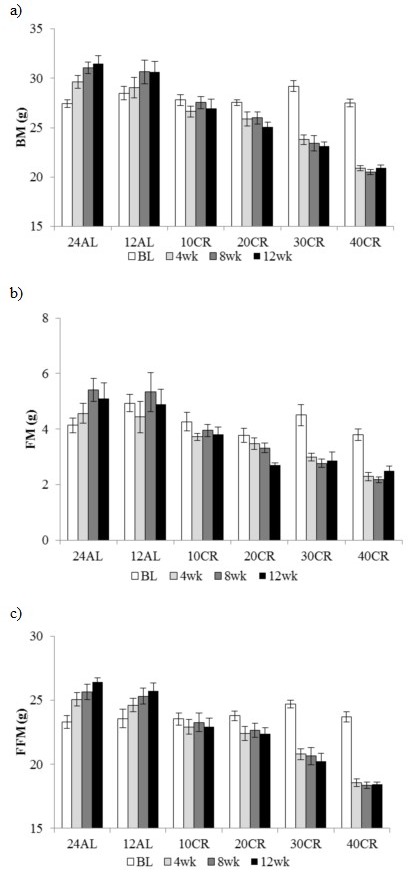
Body composition changes following 3 months of graded calorie restriction **a.** body mass (BM), **b.** fat mass (FM) and **c.** fat free mass (FFM) measure by DXA at baseline (BL), 4, 8 and 12 weeks of *ad libitum* (AL) 12 & 24h or graded levels of caloric restriction (CR) (10CR, 20CR, 30CR and 40CR), Data represented as mean ± SEM (g).

Using DXA, body mass, fat mass and fat-free mass were recorded at baseline, 4, 8 and ~ 12 weeks after CR started. By pooling all measurements there was a significant effect of time (F_(3, 120)_ = 51.96, 7.34, 23.3, *p* < 0.001), and treatment (F_(5, 40)_ = 23.16, 13.67, 19.53, *p* < 0.001) with a strong time by treatment interaction (F_(15, 120)_ = 46.55, 3.49, 20.30, *p* < 0.001) for all 3 parameters (GLM with RM for body mass, fat mass and fat-free mass respectively) (Figure 2a, 2b, 2c). There were no significant differences between any of the groups for any of the parameters at baseline. The body mass increase in the 12AL and 24AL groups was reflected by an increase in fat-free mass (2.15 ± 0.58 g, *t*-Test, t_14_ = 3.72, *p* < 0.01 and 3.32 ± 0.45 g, t_14_ = 7.31, *p* < 0.001 in 12AL and 24AL respectively) (Figure 2c) but not fat mass (Figure 2b). The reduction in body mass observed by 4 weeks in the 30 and 40CR mice was reflected by losses in both fat mass (−1.65 ± 0.42g, t_14_ = 3.96, *p* < 0.01 and −1.31 ± 0.35g t_14_ = 3.74, *p* < 0.01 respectively) and fat-free mass (−4.43 ± 0.51g, t_14_ = 8.62, *p* < 0.001 and −5.26 ± 0.42g, t_14_ = 12.42, *p* < 0.001 respectively) (Figure 2b, 2c). While no significant changes were observed in fat mass and fat-free mass by DXA in the 10 CR group throughout the three month period, the 20 CR mice reduced fat mass (−1.11 ± 0.32 g, t_14_ = 3.41, *p* < 0.05) and fat-free mass (−1.29 ± 0.41 g, t_14_ = 3.11, *p* < 0.05) after 4 weeks. Consistent with the time courses for body mass throughout the entire experiment (Figure 1) there were no further significant changes in body composition after the 4 week measurement point for any of the CR groups.

#### Body composition by dissection after three months CR

**Figure 3:**
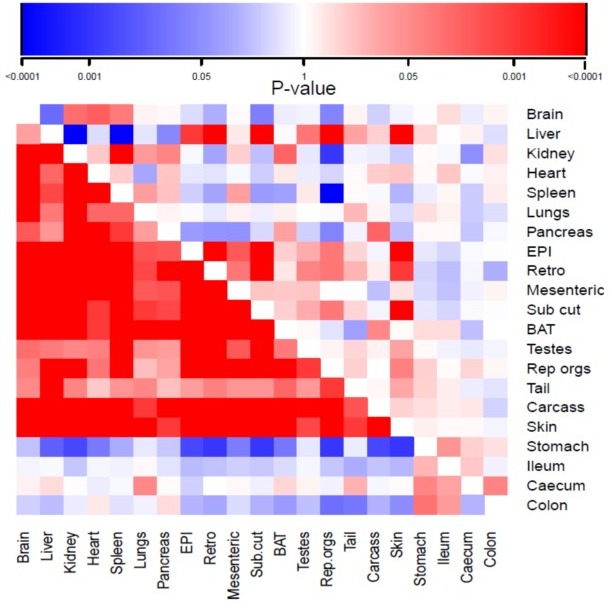
Correlation matrix showing the magnitude of the correlation in the responses to calorie restriction (CR) of different organs across all individuals The lower triangle represents animals under CR and the upper triangle animals under protein restriction (PR). EPI is epididymal white adipose tissue (WAT), Retro is retroperitoneal WAT, Sub Cut is subcutaneous WAT, rep orgs are the reproductive accessory organs, BAT is the interscapular brown adipose tissue. The scale for the correlations is shown at the top of the diagram increasing intensity of red was greater positive correlation and increasing intensity of blue was greater negative correlation (for actual correlation values see Supplementary Table 2).

The mean and standard deviations of the weights of all the wet organs at dissection across all the groups, and the calculated percent change relative to the 12AL group are provided in Table 1. The correlation coefficients of the responses of the different tissues across all the individuals are provided in Supplementary Table 2. These correlations are illustrated in Figure 3 (lower half), and show a major division in the correlation structure between the components of the alimentary tract and the other organs. The responses of the alimentary tract components were weakly positively correlated with each other, but were either weakly (ileum, colon and caecum) or strongly (stomach) negatively correlated with all the other organs. In contrast, the responses of the other organs were mostly strongly correlated with each other. The main exceptions to this were the testes, reproductive accessory organs and tail, which showed only weak correlations with the other components and between themselves.

**Table 1:** The wet tissue weights (g) of all organs across all treatment groups at the end of 3 months of calorie restriction (CR)

DIET	24AL	12AL	10CR	20CR	30CR	40CR
Carcass	15.11±0.664	14.73±1.327	13.36±1.283	12.71±1.049	11.06±0.746	10.02±0.853
%	+2.58		−10.68%	−13.71%	−24.91%	−31.97%
Stats	A	A	B	B	C	D
Skin	4.263±0.456	4.043±0.570	3.584±0.527	3.304±0.337	2.895±0.133	2.676±0.161
%	+5.44%		−11.35%	−24.95%	−28.39%	−33.81%
Stats	A	AB	BC	CD	DE	E
Tail	0.718±0.126	0.714±0.068	0.722±0.106	0.660±0.078	0.673±0.086	0.637±0.066
%	+0.5%		+1.12%	−7.56%	−5.74%	−10.78%
Stats	A	A	A	A	A	A
Brain	0.466±0.017	0.454±0.014	0.464±0.013	0.454±0.016	0.439±0.013	0.423±0.010
%	+2.64%		+2.20%	0%	−3.3%	−4.4%
stats	A	AB	A	AB	BC	C
Liver	1.146±0.212	1.240±0.201	1.023±0.117	0.8978±0.151	0.922±0.073	0.897±0.107
%	−7.58%		−17.5%	−27.60%	−25.64%	−27.66%
Stats	A	A	AB	B	B	B
Kidneys	0.445±0.069	0.433±0.101	0.357±0.069	0.325±0.105	0.268±0.018	0.230±0.015
%	+2.77%		−17.55%	−24.94%	−38.11%	−46.88%
Stats	A	AB	AB	BC	CD	D
Lungs	0.199±0.019	0.186±0.022	0.183±0.032	0.175±0.022	0.164±0.016	0.165±0.035
%	+6.99%		−1.61%	−5.91%	−11.83%	−11.29%
Stats	A	AB	AB	ABC	BC	C
Heart	0.181±0.031	0.166±0.028	0.151±0.025	0.144±0.019	0.139±0.007	0.130±0.011
%	+9.04%		−9.04%	−13.25%	−16.26%	−21.69%
Stats	A	AB	ABC	BC	CD	D
Spleen	0.114±0.051	0.089±0.033	0.070±0.008	0.072±0.026	0.046±0.019	0.03±0.008
%	+28.0%		−21.34%	−19.1%	−48.21%	−66.3%
Stats	A	A	AB	AB	BC	C
Pancreas	0.18±0.046	0.161±0.035	0.129±0.023	0.140±0.026	0.144±0.042	0.118±0.019
%	+11.80%		−19.87%	−13.04%	−10.55%	−26.71%
Stats	A	A	AB	AB	AB	B
Epi	0.773±0.267	0.831±0.401	0.535±0.221	0.412±0.110	0.220±0.084	0.174±0.216
%	−6.97%		−35.62%	−50.42%	−73.53%	−79.06%
Stats	AB	A	AB	B	C	D
Sub Cut	1.459±0.363	1.357±0.541	1.005±0.314	0.822±0.152	0.598±0.108	0.416±0.131
%	+7.52%		−25.94%	−39.42%	−55.93%	−69.34%
Stats	A	AB	BC	CD	DE	E
Retro	0.273±0.159	0.269±0.129	0.131±0.043	0.091±0.042	0.074±0.017	0.039±0.013
%	+1.49%		−51.30%	−66.17%	−72.49%	−85.50%
Stats	A	A	B	B	B	C
Mes Fat	0.533±0.137	0.441±0.145	0.356±0.047	0.307±0.061	0.268±0.069	0.179±0.046
%	+20.86%		−19.27%	−30.39%	−39.23%	−59.41%
Stats	A	AB	BC	BC	CD	D
BAT	0.201±0.039	0.189±0.046	0.146±0.036	0.136±0.015	0.127±0.019	0.105±0.020
%	+6.35%		−22.75%	−28.04%	−32.8%	−44.44%
Stats	A	AB	BC	CD	CD	D
Rep orgs	0.614±0.233	0.776±0.176	0.487±0.099	0.378±0.065	0.393±0.147	0.287±0.069
%	−20.87%		−37.24%	−51.29%	−49.35%	−63.01%
Stats	A	AB	BC	CD	CD	D
Testes	0.189±.033	0.212±0.013	0.192±0.039	0.194±0.021	0.191±0.017	0.151±0.018
%	−10.84%		−9.43%	−8.49%	−9.91%	−28.77%
Stats	A	A	A	A	A	B
Stomach	0.151±0.013	0.130±0.012	0.152±0.021	0.185±0.032	0.173±0.023	0.169±0.011
%	+16.15%		+16.92%	+42.31%	+33.1%	+30.0%
Stats	AB	A	AB	C	BC	BC
Ileum	0.636±0.112	0.596±0.087	0.516±0.141	0.663±0.160	0.615±0.161	0.685±0.122
%	+6,71%		−13.42%	+11.12%	+3.18%	+14.93%
Stats	A	A	A	A	A	A
Caecum	0.08±0.023	0.067±0.008	0.058±0.017	0.063±0.019	0.071±0.009	0.084±0.034
%	+19.40%		−13.43%	−5.97%	+5.97%	+25.37%
Stats	A	A	A	A	A	A
Colon	0.115±0.035	0.111±0.015	0.105±0.007	0.128±0.022	0.130±0.018	0.131±0.028
%	+3.60%		+5.71%	+15.32%	+17.12%	+18.02%
Stats	AB	AB	B	AB	AB	A

24AL refers to 24h ad libitum feeding. 12AL refers to *ad libitum* feeding for just 12h per day. 10CR, 20CR, 30CR and 40CR refer respectively to 10%, 20%, 30% and 40% restriction relative to their own baseline intakes. The calculated weight difference as a percentage of the weight of the same organ in the 12AL group are shown below the absolute values, % =[(group mean/12AL group mean) −1]*100. Stats refer to differences detected between groups using Tukey tests after GLM (see also Table 2). Groups sharing the same letter are nsd. Data are mean ± sd.

**Figure 4:**
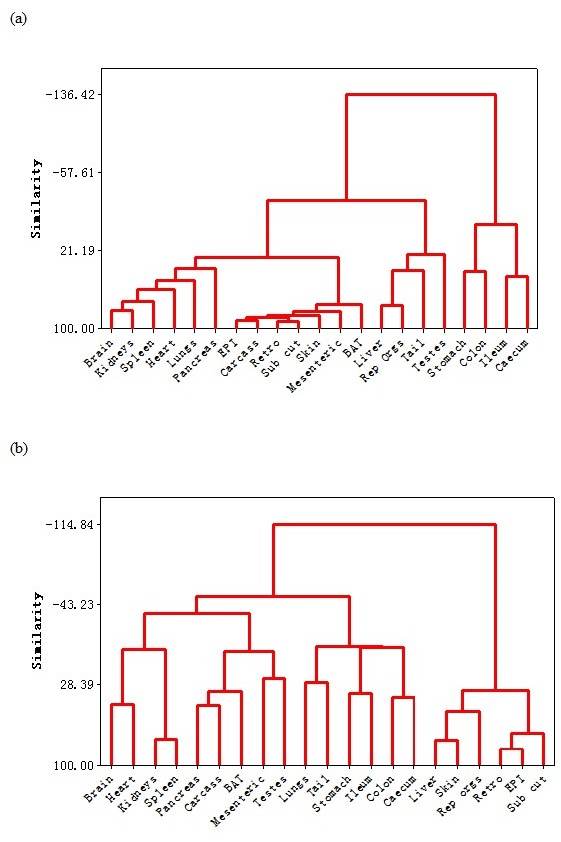
**Dendrograms showing the similarity in responses of the different organs to a.** graded caloric restriction (CR) and **b.** graded protein restriction (PR). EPI is epididymal white adipose tissue (WAT), Retro is retroperitoneal WAT, Sub Cut is subcutaneous WAT, rep orgs are the reproductive accessory organs, BAT is the interscapular brown adipose tissue.

Rep organs = reproductive organs, BAT = brown adipose tissue, Mes fat = mesenteric fat, Retro = retroperitineal, sub cut = subcutaneous fat, Epi = epididymal,

A dendrogram based on these correlation patterns is shown in Figure 4a. This analysis grouped the organ responses together into 4 major groups. Consistent with the correlation matrix (Figure 3), the first major separation was between the four components of the alimentary tract and the rest of the body. Among the organs in the rest of the body, the dendrogram analysis separated the responses into three different groups. The first group consisted of the main vital organs (brain, kidneys, spleen, heart, lungs and pancreas). The second group consisted of a mix of structural organs (carcass and skin) and the adipose tissue compartments (epididymal (EPI), retroperitoneal (retro), subcutaneous (sub cut), mesenteric and brown adipose tissue (BAT)). The final grouping involved four organs that were not functionally related. These included the reproductive tissues (accessory organs and testes), the liver and the tail.

**Figure 5:**
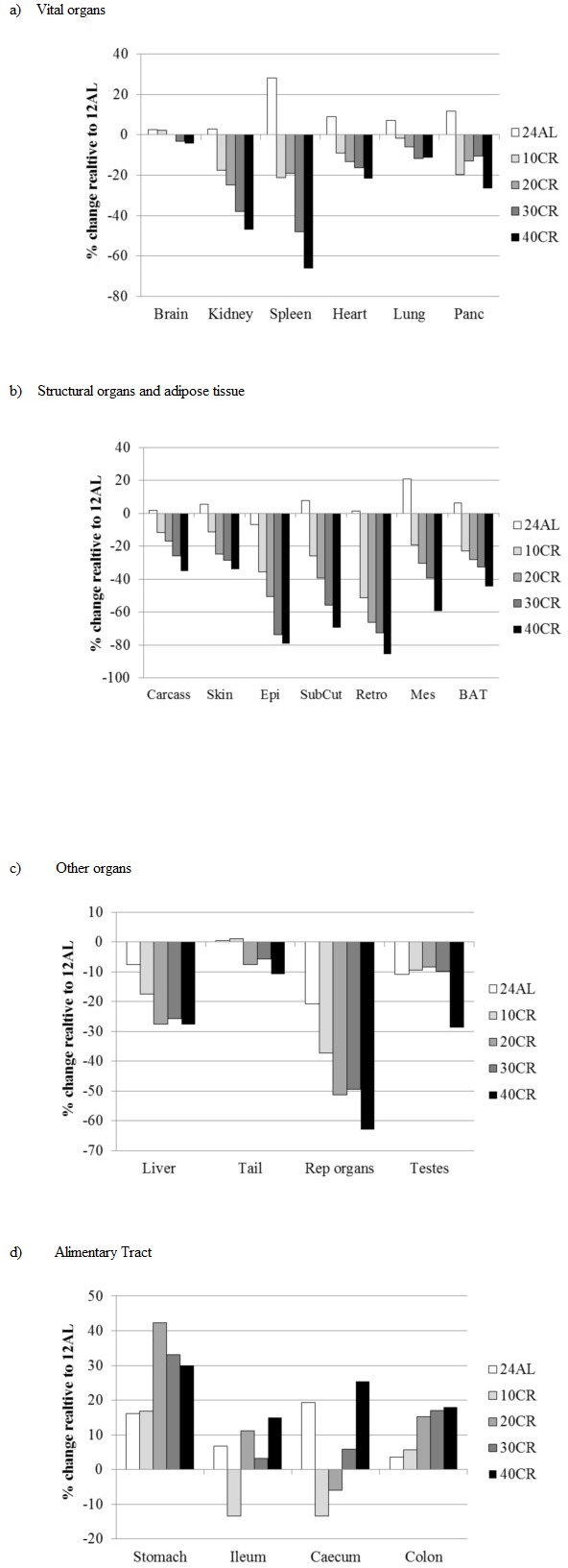
Changes in organ size following three months of graded calorie restriction (CR) Histograms showing the extent of change in organ sizes relative to the sizes of the organs in the 12AL group (% difference) after three months of CR. Tissues are grouped in accord with the hierarchical clustering analysis in Figure 4a. panc is pancreas, EPI is epididymal white adipose tissue (WAT), Retro is retroperitoneal WAT, Sub Cut is subcutaneous WAT, Mes is mesenteric WAT, rep orgs are the reproductive accessory organs, BAT is the interscapular brown adipose tissue. Compare to Figure 10 for the same changes under PR.

The patterns of change in these different organs separated into the groups identified from the correlation and dendrogram analysis are illustrated in Figure 5. The vital organs generally showed an almost linear pattern of decline in size in relation to the severity of restriction (Tables 1 and 2 and Figure 5a). However the percentage reductions relative to the 12AL group were relatively modest, especially for the brain which declined by a maximum of 4.4% under 40CR, and generally only reached significance at the highest levels of restriction (Table 1). The lungs, heart and pancreas showed larger maximal percent weight losses between 11.7 and 26.7% and the largest losses relative to the 12AL animals were observed in the kidney (maximal loss 46.9%) and spleen (66.3%). The patterns of change in the second grouping from the correlation analysis were also roughly linear with the extent of restriction (Table 1 and Figure 5b), but in these cases the extent of loss in mass relative to the 12AL group was much greater (Tables 1 and 2 and Figure 5b), particularly for the white adipose tissue components which showed maximal losses at 40CR between 59.4% (mesenteric) and 85.5% (retro). The structural components had relatively lower losses compared to the white adipose tissue, being maximally around 34% loss in the 40CR group relative to 12AL in both the skin and carcass. BAT showed an intermediate maximal relative loss of 44%. In the third group identified by the correlation analysis the changes did not appear to be linearly related to the extent of restriction. Hence the liver appeared to lose mass at a relatively constant 26% below the 12AL liver mass in the 20CR, 30CR and 40CR groups (all significantly different to 12AL but not different from each other: Table 1). The tail lost very little mass, averaging 5.7 to 10.8% below the 12AL group in the 20CR, 30CR and 40CR groups (all non-significantly different from 12AL: Tables 1 and 2), and the testes were on average 9% smaller than the 12AL group for 10CR, 20CR and 30CR groups (all non-significant), only showing a significant decline at 40CR (28.8% lower than the 12AL group). The only organs in this group that deviated from this pattern were the reproductive accessory organs, which showed a more linear decline with the extent of restriction (Tables 1 and 2 and Figure 5c). The final grouping was the alimentary tract components. These all had positive changes relative to the 12AL group. Although the patterns with change in the level of restriction were less clear (Table 1 and Figure 5d) there did appear to overall be greater investment as the extent of restriction increased.

**Table 2:** Effects of treatment (3 months exposure to calorie restriction (CR) or *ad libitum* (AL) intake) and the effect of baseline body mass on the tissue masses of C57BL/6 mice

Organ	Treatment		Baseline Body mass	
	F	p	F	p
Brain	**11.96**	**<0.0005**	0.02	0.88
Kidneys	**19.3**	**<0.0005**	7.69	0.008
Heart	**6.87**	**<0.0005**	6.17	0.017
Spleen	**18.2**	**<0.0005**	0.37	0.548
Lungs	**5.16**	**0.001**	3.63	0.064
Pancreas	**4.55**	**0.002**	1.54	0.221
Skin	**22.6**	**<0.0005**	**17.86**	**<0.0005**
Carcass	**50.79**	**<0.0005**	**16.81**	**<0.0005**
BAT	**12.78**	**<0.0005**	1.53	0.223
EPI	**30.94**	**<0.0005**	0.2	0.661
Retro	**35.71**	**<0.0005**	1.2	0.28
Sub cut	**25.29**	**<0.0005**	1.27	0.267
Mesenteric	**21.13**	**<0.0005**	1.01	0.322
Testes	**5.61**	**<0.0005**	0.1	0.757
Rep orgs	**14.63**	**<0.0005**	1.8	0.187
Liver	**7.83**	**<0.0005**	7.65	0.008
Tail	1.41	0.242	8.01	0.007
Stomach	**9.06**	**<0.0005**	0.91	0.345
Ileum	1.78	0.138	0.31	0.578
Caecum	0.66	0.658	0.02	0.894
Colon	3.79	0.006	5.49	0.024

**Data were analyzed using general linear modelling with baseline body mass as a covariate**. Bold highlighted values were significant after Bonferroni correction (p < 0.0024).

**Figure 6:**
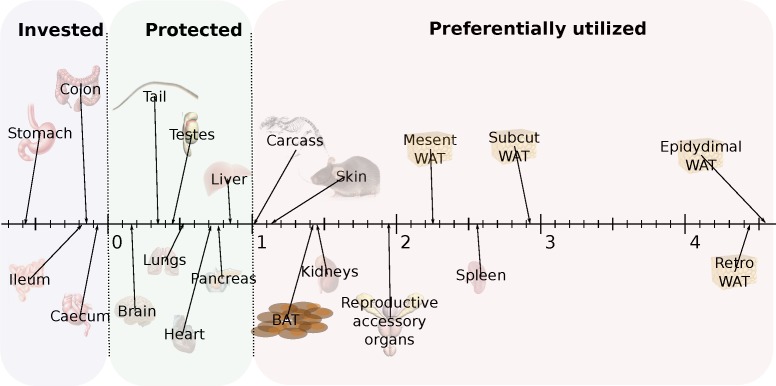
Hierarchy of utilization of different organs following three months of caloric restriction (CR) Hierarchy is reflected in the gradient of the relationship between the Log_e_ tissue weight and the Log_e_ final body mass (BM) (see Table 3 for actual regression coefficients). Tissues with values below 0 were invested in. Those with values between 0 and 1 were relatively protected. Those with values greater than 1 were preferentially utilized. Preferential utilization was greater at greater values.

Utilizing ratios to express data may lead to interpretation issues, e.g. [33]. To express the relative utilization of different tissues across all the treatment groups, avoiding the use of ratios, we plotted the final weight of each organ against the final body mass across all individuals. We then fitted a linear least squares fit regression to these data and used the gradient (β) to express the differential utilization of the tissues. On this basis, an organ with a gradient of β = 1 would be utilized at the same rate as total body mass as restriction severity increased. Anything with a gradient greater than 1 was declining more rapidly than body mass and hence was being preferentially utilized at high levels of restriction. In contrast, organs that had β gradients between 0 and 1 were being relatively protected, and any organ with a negative gradient (β < 0) was increasing in weight as the animals overall got lighter. Hence, these organs were being invested in. The parameters of the fitted regressions are shown in Table 3 and the regression gradients are illustrated in Figure 6. These data show that the organs of the alimentary tract were invested in (β < 0). This was particularly so for the stomach, but to a lesser extent for all the gut components. The organs and tissues that were relatively protected included all the vital organs such as the brain, lungs, heart, liver and pancreas. This ‘protected’ group of organs also included the tail (which was almost as protected as the brain) and the testes. Among the tissues that were preferentially utilized were the kidneys, spleen and the reproductive accessory organs. All the other preferentially utilized tissues were structural (carcass and skin) or fat depots. The most preferentially utilized fat stores were the EPI and retro.

**Table 3:** Parameters of regression equations between individual organ masses at final dissection and the final total body weight pooled across all individuals in the calorie restricted treatment groups

	Regression equation				
Organ	Intercept	Gradient	r^2^	F	P
Brain	−1.4101	0.1878	0.475	43.49	<0.0005
Kidneys	−5.8245	1.4482	0.785	167.8	<0.0005
Heart	−4.1906	0.7075	0.484	45.12	<0.0005
Spleen	−11.0757	2.5479	0.636	83	<0.0005
Lungs	−3.4186	0.5157	0.394	31.55	<0.0005
Pancreas	−4.537	0.7859	0.364	27.92	<0.0005
Skin	−2.416	1.1204	0.873	324.3	<0.0005
Carcass	−1.1831	1.0454	0.952	942.8	<0.0005
BAT	−6.5788	1.4276	0.706	113.9	<0.0005
EPI	−15.809	4.5488	0.835	238.3	<0.0005
Retro	−16.578	4.424	0.867	306.2	<0.0005
Sub Cut	−9.629	2.907	0.847	260.4	<0.0005
Mesenteric	−8.2974	2.2485	0.829	229.1	<0.0005
Testes	−3.2016	0.4664	0.233	15.28	<0.0005
Rep orgs	−7.0969	1.9385	0.575	64.58	<0.0005
Liver	−2.661	0.8216	0.538	55.78	<0.0005
Tail	−1.5324	0.354	0.197	12.52	0.001
Stomach	−0.6174	−0.378	0.141	8.7	0.005
Ileum	0.141	−0.199	0.021	0.98	0.327
Caecum	−2.4038	−0.098	0.006	0.26	0.611
Colon	−1.5546	−0.171	0.033	1.57	0.217

#### Assimilation efficiency

**Figure 7:**
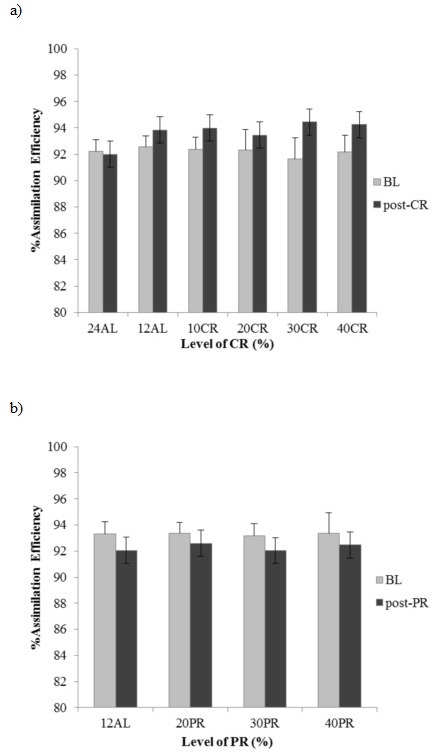
Changes in assimilation efficiency (%) following calorie restriction (CR) or protein restriction (PR) assimilation efficiency% of the different treatment groups measured during baseline (BL) and **a.** post-CR or **b.** post-PR. 24AL and 12AL refer to the two *ad libitum* groups and the 10CR, 20CR, 30CR and 40CR refer to 10, 20, 30 and 40%. 20PR, 30PR and 40PR refer to equivalent PR groups.

Assimilation efficiency calculated as the percent of the ingested energy that was assimilated, excluding urinary losses, did not differ significantly between the CR treatment groups at baseline when they were all treated identically (F_(5, 36)_ = 0.42, *p* > 0.05). Assimilation efficiency averaged 92.3 % (Figure 7a). Following CR exposure there was a significant group effect on assimilation efficiency (F_(5, 32)_ = 9.53, *p* < 0.0005) (Figure 7a). Although the assimilation efficiency appeared to increase with the level of restriction Tukey pairwise comparisons indicated that in fact all the CR groups did not differ significantly (*p* > 0.05) from each other or from 12AL, but they were all greater than the 24AL group. The extent of increase in assimilation efficiency varied between 1.16% (sd = 1.15) in the 20CR group and 2.97% (sd = 2.10) in the 30CR group. If we use the average increased assimilations, then actual realized levels of restriction in the nominally 10CR, 20CR, 30CR and 40CR groups based on gross intake were 8.4%, 18.8%, 27.0% and 38.3% respectively. At an individual level, we found no relationship between the level of assimilation efficiency when under CR and the sizes of the components of the alimentary tract (multiple regression analysis *p* > 0.05 for all alimentary tract components).

#### Bone characteristics

No restriction effect was found in the DXA measurements of bone mineral content and density (BMC and BMD) and bone area (BA). However changes over time within the diet groups were significant (GLM-RM, BMC: F_(3, 129)_ = 4.35, *p* < 0.01, BMD: F_(3, 129)_ = 3.461, *p* < 0.05, BA: F_(3, 129)_ = 13.27, *p* < 0.001). Contrary to previous reported negative impacts of CR on bone, post hoc comparisons at 12 weeks found BMC and BA to be higher (*p* < 0.05) in the 40CR mice compared to 12AL. A CR effect was found on the length of both the tibia (ANOVA, F_­(5, 39)_ = 3.69, *p* < 0.01) and femur (F_­(5, 39)_ = 5.80, *p* < 0.001) and diameter (F_­(5, 39)_ = 6.83, *p* < 0.001) of the latter only. The femur was longer in the 10, 20 and 30CR mice, significantly so in 10 and 30CR groups (16.84 ± 0.12 mm and 16.79 ± 0.29 mm, *p* < 0.05) compared to the 12AL (16.03 ± 0.11 mm), while the tibia was longest in the 20CR (18.09 ± 0.18mm, *p* < 0.01). Both the widest (2.33 ±0.06 mm) and narrowest (1.6 ±0.08 mm) diameter of the femur were also largest in the 20CR mice compared to 12 AL (2.04 ±0.03 mm and 1.33 ±0.06 mm). Although a significant response to CR was found in the dimensional measurements, no differences were found in the mechanical properties measured using the 3-point bending test. No changes in organic or mineral content of either tibia or femur were observed following CR. Analysis of the micro-architectural structure found the fractional bone volume, (i.e. the percentage of bone volume relative to the total volume (BV/TV), trabecular thickness (Tb Th) and trabecular number TbN of both the tibia and femur were higher but not significantly so in the 30CR compared to the 12AL (*t*-test, *t* = *p* > 0.05) which supports data indicating a higher BMC recorded by DXA in the CR groups.

### Protein restriction (PR)

#### Food intake

**Figure 8:**
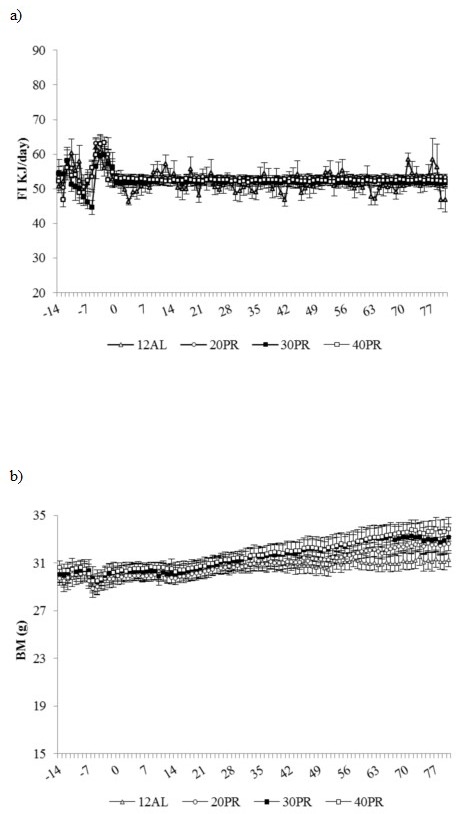
Food intake and body mass changes recorded over 3 months graded protein restriction Daily records of **a.** food intake (kJ/day), **b.** body mass (BM) (g) recorded over 2 weeks of baseline monitoring (days −14 to −1) and 12 weeks of treatment: 12h *ad libitum* (12AL) feeding and graded levels of protein restriction (PR) from 20 to 40%. Data are presented as daily mean ± SEM (g). The plots use the same scale as the plots in Figure 1 to facilitate comparison to the changes under CR.

Average food intake over the baseline period, 54.21 ± 3.91 kJ/day, was not significantly different between groups. Over the restriction period all diet groups lowered food intake (GLM-RM, time F_(1, 56)_ = 26.70, *p* < 0.001) but no differences were found between diets (Figure 8a).

#### Body mass

A similar body mass between groups was measured at the start of the treatment period, 30.11 ± 0.30g (Figure 8b). Although the level of PR did not affect body mass over the restriction phase (GLM-RM, diet F_(3, 56)_ = 0.3, *p* > 0.05), there was a time effect (GLM-RM, F_(1, 28)_ = 129.88, *p* < 0.001) with a significant interaction between time and diet (GLM-RM, F_(3, 28)_ = 3.47, *p* < 0.05) and a significant but similar increase in body mass found in all groups (Figure 8b).

#### Analysis of body composition by DXA

**Figure 9:**
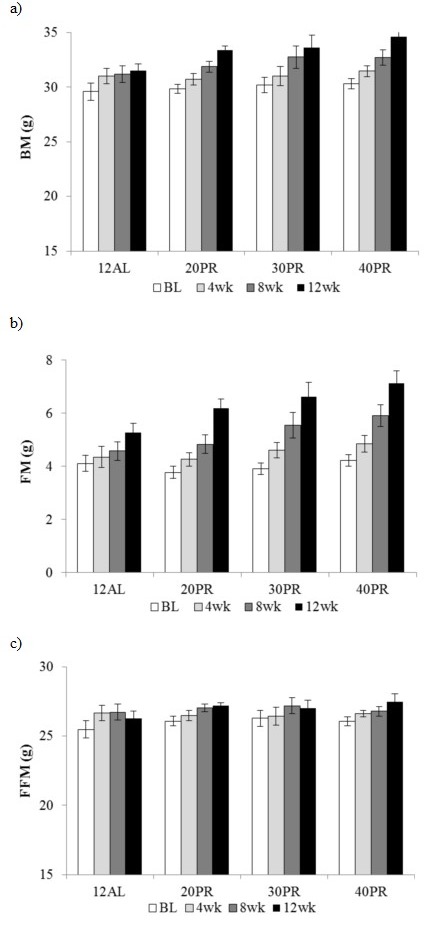
Body composition changes following 3 months of graded protein restriction **a.** body mass (BM), **b.** fat mass (FM) and **c.** fat free mass (FFM) measured by DXA at baseline (BL), 4, 8 and 12 weeks of 12h *ad libitum* (12AL) or graded levels of protein restriction (PR) (20PR, 30PR and 40PR). Data represented as mean ± SEM (g).

A time, but no diet effect, with a significant interaction was found over the 4 weekly measures of body mass (GLM-RM, time F_(3, 84)_ = 80.56, *p* < 0.001, interaction F_(9, 84)_ = 2.92, *p* < 0.01) and fat mass (GLM-RM, time F_(3, 84)_ = 105.30, *p* < 0.001, interaction F_(9, 84)_ = 3.45, *p* < 0.001). Fat-free mass however was only affected by time (GLM-RM, time F_(3, 84)_ = 15.44, *p* < 0.001) (Figure 9a, 9b and 9c).

#### Body composition by dissection at three months

The mean and standard deviations of the weights of all the wet organs at dissection across all the groups are provided in Table 4. The correlation coefficients of the responses of the different tissues across all the individuals are provided in the supplementary material (supplementary Table 2). These correlations are illustrated in Figure 3 in the top half of the diagram. The correlation structure under PR was completely different from that under CR. The correlations between tissues were much weaker than under CR and in both positive and negative directions. Moreover, there was no clear separation between the responses of the alimentary tract components and the rest of the body as was evident under CR. The dendrogram (Figure 4b) based on this correlation matrix separated the responses into four groups. Note that the branch points in this dendrogram sit much further back than in the CR dendrogram (Figure 4a), reflecting the poorer correlations between tissues in the PR animals. The first group comprised four vital organs (brain, heart, kidneys and spleen). The second group comprised 5 tissues with variable functions (carcass, BAT, mesenteric fat, testes and the pancreas). The third group comprised all the alimentary tract components along with the tail and lungs. Finally, the fourth group consisted of three white adipose tissue depots along with the liver, skin and reproductive organs.

**Table 4:** Wet tissue weights (g) of all organs across all treatment groups at the end of 3 months of protein restriction (PR)

DIET	12AL	20PR	30PR	40PR
Carcass	15.48±2.80	16.82±3.70	16.55±3.00	16.11±3.15
%		+8.65%	+4.55%	+4.06%
Skin	4.423±0.330	4.826±0.260	4.707±0.693	4.832±0.455
%		+9.1%	+6.42%	+9.24%
Tail	0.669±0.054	0.676±0.028	0.659±0.028	0.679±0.033
%		+1.04%	−1.49%	+1.49%
Brain	0.466±0.017	0.466±0.019	0.459±0.009	0.462±0.024
%		0	−1.46%	−0.88%
Liver	1.157±0.270	1.260±0.193	1.305±0.134	1.307±0.222
%		+8.9%	+12.79%	+12.96%
Kidneys	0.434±0.074	0.423±0.075	0.403±0.028	0.417±0.044
%		−2.53%	−7.14%	−3.9%
Lungs	0.301±0.034	0.313±0.058	0.317±0.043	0.317±0.037
%		+3.98%	+5.31%	+5.31%
Heart	0.177±0.015	0.185±0.027	0.175±0.021	0.174±0.028
%		+4.52%	−1.13%	−1.69%
Spleen	0.098±0.029	0.098±0.030	0.078±0.028	0.094±0.044
%		0	−20.4%	−4.1%
Pancreas	0.28±0.098	0.282±0.108	0.251±0.048	0.293±0.048
%		+0.7%	−10.4%	+4.6%
Epi	0.951±0.351	0.983±0.171	1.184±0.442	1.314±0.357
%		+3.4%	+24.5%	+38.2%
Sub Cut	1.592±0.330	1.854±0.542	2.111±0.638	2.224±0.633
%		+16.4%	+32.6%	+39.7%
Retro	0.359±0.169	0.434±0.153	0.446±0.149	0.518±0.187
%		+20.9%	+24.2%	+44.3%
Mesenteric	0.383±0.136	0.407±0.144	0.435±0.163	0.517±0.116
%		+6.3%	+13.6%	+35.0%
BAT	0.226±0.052	0.218±0.021	0.234±0.050	0.228±0.066
%		−3.5%	+3.5%	+0.8%
Rep orgs	0.646±0.103	0.709±0.137	0.809±0.176	0.763±0.176
%		+9.75%	+25.2%	+18.1%
Testes	0.207±0.022	0.223±0.021	0.208±0.014	0.234±0.031
%		+7.7%	+0.5%	+13.0%
Stomach	0.203±0.026	0.185±0.048	0.190±0.015	0.188±0.021
%		−8.9%	−6.4%	−7.4%
Ileum	0.949±0.181	0.834±0.093	0.820±0.119	0.815±0.113
%		−12.11%	−13.6%	−14.1%
Caecum	0.099±0.017	0.084±0.024	0.090±0.019	0.090±0.021
%		−15.5%	−9.1%	−9.1%
Colon	0.160±0.044	0.134±0.047	0.163±0.030	0.153±0.022
%		−16.3%	+1.9%	−4.4%

12AL refers to ad libitum feeding for just 12 hours per day. 20PR, 30PR and 40PR refer respectively to 20%, 30% and 40% restriction of protein relative to their own baseline intakes. The calculated weight difference as a percentage of the weight of the same organ in the 12AL group are shown below the absolute values, % =[(group mean/12AL group mean) −1]*100. Data are mean and ± 1.0 sd. None of the differences between treatment groups were significant after Bonferroni correction.

**Figure 10:**
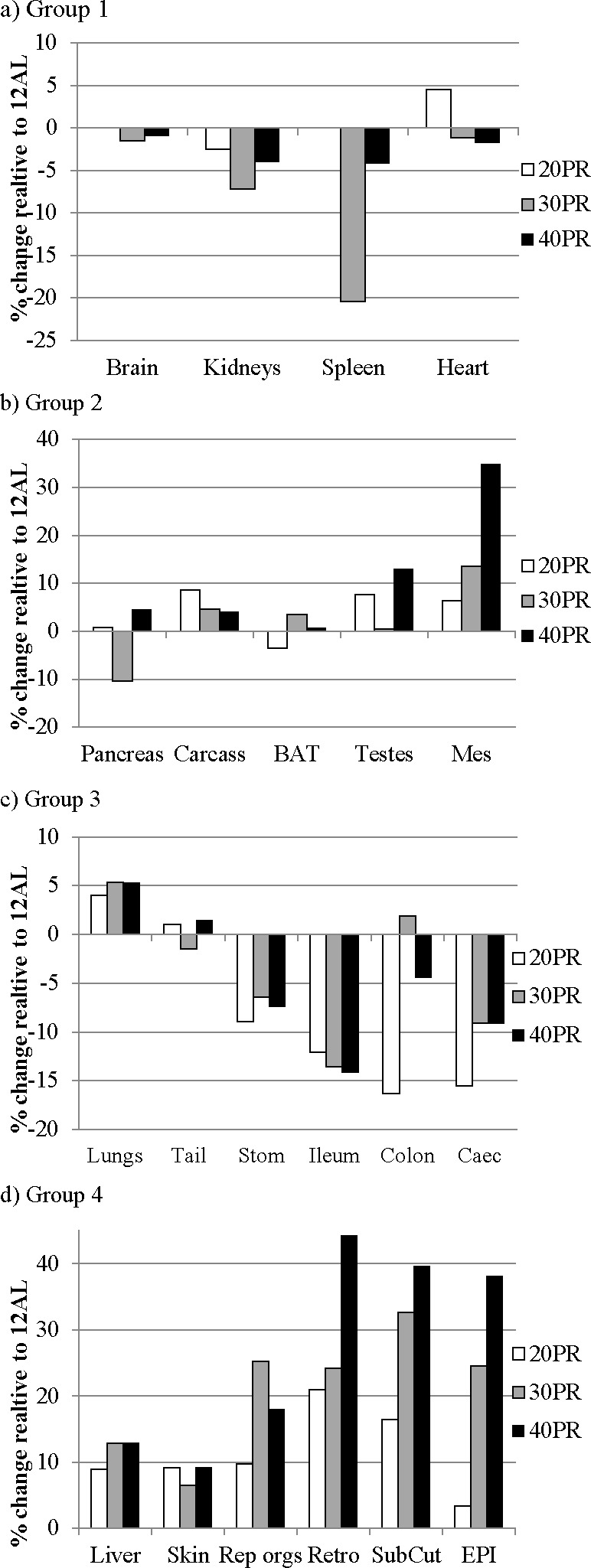
Changes in organ size following three months of graded protein restriction Histograms showing the extent of change in organ sizes after three months of protein restriction (PR) relative to the sizes of the organs in the *ad libitum* fed group (% difference). EPI is epididymal white adipose tissue (WAT), Retro is retroperitoneal WAT, Sub Cut is subcutaneous WAT, Mes is mesenteric, rep orgs are the reproductive accessory organs, BAT is the interscapular brown adipose tissue. Tissues are grouped in accord with the hierarchical clustering analysis in Figure 4b. The plots use the same scale as the plots in Figure 5 to facilitate comparison to the changes in the same tissues under CR.

The patterns of change in the individual organs in these 4 groupings are illustrated in Figure 10 and the relevant statistics are shown in Table 4. PR did not significantly affect the sizes of the different organs, apart from a marginal effect on the testes (GLM, *p* > 0.05), not significant after Bonferroni correction). There were significant relationships however between the final masses of several organs and the initial baseline body mass (Table 5). These included the major structural organs (carcass, skin and tail) and the liver.

**Table 5:** Effects of treatment (three months exposure to 3 protein restricted diets (PR) or *ad libitum* protein intake) and the effect of baseline body mass on the tissue masses of C57BL/6 mice

	Treatment		Baseline Body mass	
Organ	F	p	F	p
Brain	0.29	0.831	0.57	0.456
Liver	1.51	0.234	**10.33**	**0.003**
Heart	0.08	0.968	0.43	0.516
Kidney	0.40	0.756	0.10	0.755
Lungs	0.24	0.870	0.03	0.874
Spleen	2.00	0.137	0.01	0.932
Pancreas	0.42	0.739	3.03	0.093
Retro	1.76	0.177	3.03	0.093
EPI	2.31	0.098	3.51	0.072
Sub Cut	2.07	0.127	2.73	0.110
BAT	0.16	0.921	0.17	0.683
Mesenteric	1.47	0.245	0.39	0.538
Carcass	1.02	0.398	**15.27**	**0.001**
Skin	1.86	0.159	**11.48**	**0.002**
Tail	0.39	0.763	**21.60**	**0.001**
Rep orgs	1.74	0.182	2.24	0.146
Testes	3.46	0.030	7.59	0.010
Stomach	0.83	0.487	4.49	0.043
Colon	0.52	0.670	1.84	0.186
Ileum	1.65	0.201	7.52	0.011
Caecum	0.44	0.727	1.37	0.252

#### Assimilation efficiency

Assimilation efficiency did not differ between PR treatments at baseline, average AE 93.3% (F_(3, 28)_ = 0.387, (*p* > 0.05) or following 3 months PR treatment or following 3 months PR treatment, average AE 92.3% (F_(3, 28)_ = 0.368, *p* > 0.05) (Figure 7b).

### Comparison of responses to calorie and protein restriction (CR vs PR)

Since protein contents of the PR diets were designed to match the protein intakes of the nominal 20, 30 and 40% CR animals, we compared the body composition responses of the CR groups to the equivalent PR animals to establish the extent to which the impact of CR might be attributable to the reduced levels of protein in the CR diet (Table 6). If for example the decline in weight of a particular organ under CR was mirrored by the same change under PR then we could attribute the change in that organ to the protein rather than calorie deficit. In contrast, if the loss in weight under CR was not mirrored by the same change under PR then we could be confident the effect was not due to the protein deficit and more likely due to the calorie deficit. Both CR and PR experiments had a 12AL group fed the same diet throughout. As expected most organs in these two groups did not differ significantly (*p* > 0.0006 equivalent to > 0.05 using Bonferroni correction) between the CR and PR treatments, although we did detect significant differences in the sizes of the lungs, stomach, caecum and ileum. The sources of these differences are unclear. Comparing the 20CR and 20PR groups the organs that were significantly different (GLM, *p* < 0.0006) included the liver, lungs, reproductive accessory organs, skin, and the BAT, retro, EPI and subcutaneous fat depots. Comparing the 30CR and 30PR groups the significantly different organs included everything except the brain, testes, tail, mesenteric fat depot and the four components of the alimentary tract. Finally, comparing the 40CR and 40PR groups, everything was significantly different except the tail and the four components of the alimentary tract.

**Table 6:** Statistical comparison of organ sizes in calorie and protein restricted groups (CR and PR)

Comparison	12AL v 12AL		20CR v 20PR		30CR v 30PR		40CR v 40PR	
Organ	F	p	F	p	F	p	F	P
Brain	1.47	0.235	2.47	0.138	13.44	0.003	**19.80**	**<0.0005**
Liver	0.48	0.499	**17.70**	**<0.0005**	**44.78**	**<0.0005**	**32.55**	**<0.0005**
Heart	1.19	0.294	9.38	0.008	**18.08**	**<0.0005**	**18.91**	**<0.0005**
Kidney	1.49	0.242	4.66	0.049	**115.93**	**<0.0005**	**143.10**	**<0.0005**
Lung	**62.90**	**<0.0005**	**40.33**	**<0.0005**	**78.65**	**<0.0005**	**162.40**	**<0.0005**
Spleen	0.33	0.577	5.10	0.040	**20.21**	**<0.0005**	**65.43**	**<0.0005**
Pancreas	10.33	0.006	11.76	0.004	**43.55**	**<0.0005**	**100.50**	**<0.0005**
Testes	0.32	0.583	15.52	0.001	4.40	0.056	**47.54**	**<0.0005**
Rep orgs	3.28	0.092	**38.31**	**<0.0005**	**24.38**	**<0.0005**	**56.55**	**<0.0005**
Carcass	0.47	0.503	6.24	0.026	**20.04**	**<0.0005**	**32.97**	**<0.0005**
Skin	2.92	0.110	**102.10**	**<0.0005**	**116.46**	**<0.0005**	**178.32**	**<0.0005**
Tail	2.10	0.169	0.16	0.700	0.17	0.688	2.62	0.127
Retro	1.43	0.252	**43.18**	**<0.0005**	**42.47**	**<0.0005**	**58.99**	**<0.0005**
EPI	0.40	0.536	**62.40**	**<0.0005**	**32.01**	**<0.0005**	**102.17**	**<0.0005**
Sub Cut	1.10	0.312	**26.72**	**<0.0005**	**43.46**	**<0.0005**	**70.53**	**<0.0005**
Mesenteric	0.68	0.423	5.79	0.030	6.32	0.026	**65.18**	**<0.0005**
BAT	2.21	0.160	**70.02**	**<0.0005**	**28.32**	**<0.0005**	**28.08**	**<0.0005**
Stomach	**51.70**	**<0.0005**	0.02	0.890	3.11	0.101	5.61	0.032
Ileum	**24.61**	**<0.0005**	6.83	0.020	7.99	0.014	5.20	0.038
Caecum	**25.95**	**<0.0005**	4.93	0.043	5.30	0.038	5.09	0.039
Colon	8.86	0.010	1.15	0.302	6.65	0.023	3.08	0.100

**For data on mean and sd's of organ sizes in each group refer to Tables 1 and 4**. 12AL refers to mice that had ***ad libitum*** (AL) access to food for 12h per day. 20CR, 30CR and 40CR are mice under 20, 30 and 40% CR and 20PR, 30PR and 40PR refer to groups under 20, 30 and 40% PR respectively. Items highlighted in bold are significant after Bonferoni correction for multiple testing.

### Calculated energy equivalence of the altered tissue masses under CR

We did not measure directly the energy contents of the component tissues following dissection since we preserved the tissues for other analyses (see methods). All tissues consist of a mix of water containing no usable energy and organic materials that can be mobilized to provide energy. To convert the changes in wet tissue mass from the dissections into equivalent energy it is therefore necessary to know the tissue water contents. We measured the water contents in the tissues of 60 individual C57BL/6 mice dissected using the same protocol as used for the CR and PR animals. Tissues were dried to constant weight (14 days at 60ÐC as described previously [34]. Water contents of the vital organs and alimentary tract averaged around 70 to 72.5% and were highly reproducible (standard errors across all individuals for given tissues were generally under 1%) (Table 7). In contrast the water contents of the adipose tissue depots were highly variable between individuals (except for BAT). Across individuals there was no relationship between the size of a tissue depot and its water content so we assumed that when an animal withdrew tissue, the tissue composition was unchanged. When mobilizing the lean tissue compartments, we assumed the tissue comprised the relevant amount of water, with the balance comprising protein with an energy equivalence of 17 kJ/g [35] and that when the animals utilized their fat stores, we assumed the tissue comprised the relevant amount of water and the balance was lipid comprising 39.5 kJ/g [35]. We utilized the average difference in tissue sizes between each of the CR groups, and the 12AL group, to make these calculations and then summed the energy across all the tissues to obtain the energy made available by reducing their tissues masses. These calculations are summarized in Table 8.

**Table 7:** Percentage water contents of the tissues of C57BL/6 mice

Tissue	water content (%)	SEM
Spleen	78.0	2.36
Brain	77.6	0.77
Lungs	76.1	0.43
Heart	75.4	0.36
Kidneys	73.6	0.19
Testes + rep orgs	69.4	0.49
Liver	69.0	0.16
Pancreas	61.0	0.73
**Mean Vital organs**	**72.5**	
EPI	38.7	11.15
BAT	29.8	0.89
Sub cut	25.4	4.14
Mesenteric	23.9	5.58
Retro	17.1	3.50
**Mean Fat Tissue**	**27.0**	
Carcass	63.4	0.34
Tail	52.4	0.10
Skin	47.4	1.08
**Mean Structural**	**54.4**	
Stomach	71.8	0.26
Caecum	71.7	0.79
Ileum	70.7	1.53
Colon	68.2	0.69
**Mean Alimentary**	**70.6**	

**Data was based on dissection of 60 mice and drying tissues for 14 days at 60°C**. Data are averages across individuals and SEM.

**Table 8:** Calculated energy and protein released by reducing the sizes of the major organs when under calorie restriction (CR)

Tissue		10CR	20CR	30CR	40CR
Carcass	Mass loss	1.370	2.020	3.670	4.710
	Dry loss	0.501	0.739	1.340	1.720
	Energy (kJ)	8.52	12.56	22.78	29.24
Skin	Mass loss	0.459	0.739	1.148	1.367
	Dry loss	0.241	0.388	0.604	0.719
	Energy (kJ)	4.10	6.59	10.27	12.23
Tail	Mass loss	−0.008	0.054	0.041	0.077
	Dry loss	−0.004	0.026	0.019	0.037
	Energy (kJ)	−0.07	0.44	0.32	0.63
**Structural**	**Energy (kJ)**	**12.55**	**19.59**	**33.37**	**42.10**
**TOTAL**	**Protein (g)**	**0.74**	**1.15**	**1.96**	**2.48**
Brain	Mass loss	−0.010	0.000	0.015	0.031
	Dry loss	−0.002	0.000	0.003	0.007
	Energy (kJ)	−0.03	0.00	0.05	0.12
Liver	Mass loss	0.217	0.342	0.318	0.343
	Dry loss	0.067	0.106	0.098	0.106
	Energy (kJ)	1.14	1.80	1.66	1.80
Kidneys	Mass loss	0.076	0.108	0.165	0.203
	Dry loss	0.020	0.028	0.043	0.053
	Energy (kJ)	0.34	0.48	0.73	0.90
Lungs	Mass loss	0.003	0.011	0.022	0.021
	Dry loss	0.001	0.003	0.005	0.005
	Energy (kJ)	0.02	0.05	0.09	0.09
Heart	Mass loss	0.151	0.144	0.139	0.130
	Dry loss	0.015	0.022	0.027	0.036
	Energy (kJ)	0.26	0.37	0.46	0.61
Spleen	Mass loss	0.019	0.017	0.043	0.059
	Dry loss	0.004	0.004	0.009	0.013
	Energy (kJ)	0.07	0.07	0.15	0.22
Pancreas	Mass loss	0.032	0.021	0.017	0.043
	Dry loss	0.012	0.008	0.007	0.017
	Energy (kJ)	0.20	0.14	0.12	0.29
**Vital organs**	**Energy (kJ)**	**1.99**	**2.91**	**3.26**	**4.03**
**TOTAL**	**Protein (g)**	**0.12**	**0.17**	**0.19**	**0.24**
EPI	Mass loss	0.292	0.419	0.611	0.657
	Dry loss	0.179	0.257	0.374	0.403
	Energy (kJ)	7.07	10.15	14.77	15.92
Sub Cut	Mass loss	0.352	0.535	0.759	0.941
	Dry loss	0.262	0.400	0.566	0.702
	Energy (kJ)	10.35	15.80	22.26	27.72
Retro	Mass loss	0.138	0.178	0.194	0.230
	Dry loss	0.114	0.147	0.161	0.191
	Energy (kJ)	4.50	5.80	6.35	7.54
Mes Fat	Mass loss	0.085	0.134	0.173	0.262
	Dry loss	0.065	0.102	0.132	0.199
	Energy (kJ)	2.57	4.03	5.21	7.87
BAT	Mass loss	0.043	0.053	0.062	0.084
	Dry loss	0.030	0.037	0.043	0.059
	Energy (kJ)	1.18	1.46	1.70	2.33
**Adipose**	**Energy (kJ)**	**25.67**	**37.24**	**50.29**	**61.38**
Rep orgs	Mass loss	0.289	0.398	0.383	0.489
	Dry loss	0.088	0.122	0.117	0.149
	Energy (kJ)	1.50	2.07	1.99	2.54
Testes	Mass loss	0.020	0.018	0.021	0.061
	Dry loss	0.006	0.005	0.006	0.019
	Energy (kJ)	0.10	0.09	0.11	0.32
**Rep tissue**	**Energy (kJ)**	**1.60**	**2.16**	**2.10**	**2.86**
**TOTAL**	**Protein**	**0.09**	**0.13**	**0.12**	**0.17**
Stomach	Mass loss	−0.022	−0.055	−0.043	−0.039
	Dry loss	−0.006	−0.016	−0.012	−0.011
	Energy (kJ)	−0.11	−0.26	−0.21	−0.19
Ileum	Mass loss	0.080	−0.067	−0.019	−0.089
	Dry loss	0.023	−0.020	−0.006	−0.026
	Energy (kJ)	0.40	−0.33	−0.10	−0.44
Caecum	Mass loss	0.009	0.004	−0.004	−0.017
	Dry loss	0.003	0.001	−0.001	−0.005
	Energy (kJ)	0.04	0.02	−0.02	−0.08
Colon	Mass loss	0.006	−0.017	−0.019	−0.020
	Dry loss	0.002	−0.005	−0.006	−0.006
	Energy (kJ)	0.03	−0.09	−0.10	−0.11
**Gut**	**Energy (kJ)**	**0.37**	**−0.67**	**−0.42**	**−0.82**
**TOTAL**	**Protein (g)**	**0.02**	**−0.04**	**−0.03**	**−0.05**
**Grand total**	**Energy (kJ)**	**42.2**	**61.2**	**88.6**	**109.5**
% contribution	Structural	29.8	32.0	37.7	38.4
	Vital orgs	4.7	4.8	3.7	3.7
	Adipose	60.9	60.8	56.8	56.0
	Rep tissue	3.8	3.5	2.4	2.6
	Alimentary	0.9	−1.1	−0.5	−0.8
**Shortfall**	**Days 1-29**	135.0	305.8	439.2	623.0
% contribution	31.2	19.7	19.9	17.5
**Grand total**	**Protein (g)**	0.97	1.41	2.25	2.84
Shortfall	Days 1-29	1.63	3.64	5.23	7.42
%contribution		59.1	38.5	43.0	38.3

As the severity of CR increased the amount of energy withdrawn from body tissues also increased from 42.2 kJ at 10CR to 109.5 kJ at 40CR. The proportional contribution to these totals from different tissues was not constant. The proportion of energy supplied from structural tissues (carcass, skin and tail) increased as the severity of CR increased from 29.8 % at 10CR to 38.4% at 40CR. In contrast, the percent contributions from the vital organs, and the adipose tissue, declined as the severity of CR increased. In the case of the vital organs, the contribution fell from 4.7% to 3.7% as CR increased from 10 to 40 % and in fat tissue it fell from 60.9% at 10CR to 56% at 40CR. The reproductive organs also decreased from 3.8% to 2.6%.

We calculated the energy shortfall of the supplied food relative to the baseline intake, adjusting for the change in assimilation efficiency under restriction, over the first 29 days of restriction. We chose this period because from the DXA analysis it seemed that all the organ changes were complete after the first month and 29 days was the average inflection point of the mass loss curve. This allowed us to calculate the contribution that withdrawing tissues had made to the total energy shortfall. At 10CR this was 31.2%. However, for the more severe restrictions the contribution was remarkably similar across the different levels at 17.5 to 19.9% (Table 8). The calculated protein released by mobilizing the tissues was also estimated and then compared with the shortfall in protein intake. We compared the estimated intake of 0.668g per day protein at baseline and the estimated levels of restriction (accounting for changes in assimilation). Over the first 29 days of restriction the percent contribution of withdrawn tissue to the protein shortfall was 59.1 % in the 10CR group. In the other three groups the contribution was lower, but similar across the groups at 38 to 43%.

## DISCUSSION

### Calorie restriction

The trajectories of body mass change and the calculated inflection points of the mass loss curves suggested that independent of the level of CR, it took the animals about 29 days to adjust to the imposition of CR feeding. This was consistent with the longitudinal DXA analyses of body composition, which showed a large significant difference by day 30, but no significant further change until the end of the experiment. The detailed changes in body composition that we detected at the end of the three month feeding had probably already occurred therefore by the end of the first month of restriction. The dynamics of change during the first 30 days of restriction remain unclear from this study. However, several previous studies have explored changes in organ sizes during this early dynamic phase of restriction, and they suggest that fat withdrawal occurs rapidly [36] and that the liver is among the first organs to respond by decreasing in weight [37] before detectable changes occur in the heart and brain.

We found that mice under CR invested in growth of their alimentary tracts, particularly the stomach. Previous work has also suggested that CR induced growth of the stomach of rats, particularly the fundus [38]. Although this was not observed in other studies where stomach weight was preserved but not enlarged [30]. Similarly others found preservation but not enlargement of the stomach, small intestine and large intestine of C57BL/6 mice subjected to 27% CR at 14 months of age for 70 days [31]. The enlargement of the small intestine was significant when body mass was used as a covariate in the analysis. The enlargement reported in the current study was probably driven by the fact the mice on restriction rapidly consume all the food that is provided for them. This may be adaptive because mice in the wild under restriction may need to ingest food rapidly to avoid it being eaten by conspecific or interspecific competitors. Enlargement of the other portions of the alimentary tract was less marked than for the stomach, but consistent with previous observations of elevated protein synthesis rates in the intestines of CR rats [39, 40]. The enlargement of the alimentary tract was accompanied by an increase in assimilation efficiency for the mice under CR (relative to the 24AL group), by on average 1.7 %. Others also reported an increase in digestive efficiency by mice on 27% CR by 1.58% but in that case the difference was not significant [31]. In our study, these changes were not closely linked together, since there was no correlation at the individual level between the elevated assimilation and the morphology of the alimentary tract. More likely the elevated assimilation was linked to a combination of changes in gross morphology and changes in nutrient transport, since previous work has shown that sugar transport and the absorption of amino acids are both elevated under long term CR in mice [41, 42]. These changes together clearly moderate the impact of restriction. The energy (and protein) required to grow the alimentary tract was trivially small compared with the consequent amelioration of the restriction. Hence, on average, the change in the tract morphology required an investment of between 0.4 to 0.8 kJ of energy (Table 8), yet the improved assimilation resulted in between 0.58 and 0.89 kJ/day greater energy absorption. So the cost of growing the tract was covered by the improved energy absorption in just one day, and the benefits persisted through the restriction period.

Apart from the alimentary tract all the other body tissues lost weight under CR and increasingly so in relation to the severity of restriction. However, there was a clear hierarchy in utilization with some organs/tissues preferentially utilized while others were protected. Among the most preferentially utilized tissues were the adipose tissue depots. This preferential utilization of adipose tissue has been reported previously in several studies [28, 43-46] including in humans [47-49] and is consistent with the fact that adipose tissue contains the least water (Table 7) and lipids have the highest energy yield per gram [35]. Adipose tissue therefore represents the most effective source of energy to make up for the immediate shortfall in intake when CR commences. The utilization of fat, however, was regulated so that its use was proportional to the level of restriction. This strongly suggests that the mice do not employ a strategy of first utilizing their fat stores and then drawing on other tissues when placed under restriction. Rather they continuously coordinate the use of both fat and lean tissues from the onset of restriction in relation to the restriction level. Presumably the slightly greater reliance on fat in the 10 % CR group explains why the initial weight loss was not linearly related to the restriction level (Figure 1c).

Previous work has suggested that utilization of fat during CR may occur preferentially from the visceral adipose tissue depots leading to a change in fat distribution [46] (but see [48, 49] for studies that found no such effect). Our data were consistent with this pattern, since EPI and retro stores were preferentially exploited as CR severity increased relative to the sub cut compartment in the 12AL animals. Mesenteric fat, however, contrasted these patterns and was utilized less than the other three stores. Overall, however, the ratio of sub cut to visceral (summed EPI, retro and mesenteric fat) fell from 1.13 in 12AL animals to 0.94 in 40CR animals. This trend was consistent with the supposed remodeling away from the visceral compartment as severity of CR increased, although the ratio was almost constant in the 10, 20 and 30 % CR groups (1.017, 0.985 and 1.007 respectively).

The fact that use of fat and lean tissue is coordinated and simultaneous rather than sequential leads inevitably to the steady state levels of body fat, after the dynamic phase of restriction is completed, being in proportion to the level of restriction (Figure 5, Tables 1 and 2). Since life and health span also vary in direct relation to the level of restriction [7, 50] the effect of CR on fat storage has been suggested to be the primary mechanism by which CR exerts its life and healthspan enhancing effects [46, 51-53]. Surgically removing (particularly visceral) fat increases lifespan [53] and mice with adipose tissue selective ablation of the insulin receptor (FIRKO mice) have reduced adipose tissue and live about 18 % longer. However, applying CR to ob/ob mice results in a mouse that is still fatter than control AL fed wild type mice yet lives longer [54]. Moreover, across strains of mice that varied in the lifespan response to CR, it was those mice that lost the most fat that had the most negative lifespan responses to CR, suggesting that fat loss may be actually detrimental to the CR effect [55]. However if the analysis of these data is restricted to mice that improved lifespan when treated with CR, this negative relationship disappears [2], although it does not become positive. The most preserved adipose tissue depot was the interscapular BAT. Nevertheless it was still preferentially utilized relative to other organs (gradient > 1) and in the 40CR group the BAT was only 44.4% of that in the 12AL animals. Very few other studies have examined the effects of CR on BAT, an exception being Selman (2005) who found in rats that BAT was much larger in the CR animals after both 6 and 24 months of restriction [30]. We did not replicate these findings in mice after three months of restriction.

Consistent with many other studies on calorie reduced diets we observed that there was also a substantial reduction in lean tissue mass. In fact, although the proportional use of lean tissue (ie non adipose tissue but excluding the vital and reproductive organs) was lower than the adipose tissue stores (Figure 6) the absolute weight loss in the carcass and skin was much higher. For example, compared to 12AL mice, the mice on 40CR lost 2.1 g of fat across the four white adipose tissue stores but lost 4.7 g from the carcass and 1.4 g from the skin. This reduction contrasts with early studies, which suggested that lean tissue mass is preserved under CR [37]. An even greater difference was observed after six months of CR in rats, where the carcass was 30 g lighter and skin 10g lighter but the combined fat stores only differed by just under 5 g [31]. It might be argued that the failure to preserve lean tissue mass occurred because under the protocol we used protein was also restricted at the same time as energy [56]. This interpretation seems unlikely, however, because when we placed mice under PR without a calorie deficit, the same reductions in the lean tissue mass did not occur (Figure 10 and Table 4). The lean tissue reductions were therefore driven by the shortfall in calories rather than protein. In fact energy in these tissues contributed between 29.8 and 38.4 % of the total energy released by tissue mobilization, and this percentage increased as the severity of restriction increased, while the percent contribution from the fat stores declined. The reduction in lean tissue mass was therefore a substantial contributor to the energy shortfall.

We found that most of the vital organs also lost weight in relation to the extent of restriction, but they were relatively protected (i.e. the gradients of mass at dissection against total mass were < 1.0) (Table 3 and Figure 6). The main exceptions were the kidneys, spleen and reproductive accessory organs where the gradients all exceeded 1.0, indicating preferential utilization. Preferential utilization of the reproductive accessory organs was not surprising given that one idea for the life enhancing impact of CR is that it entails a diversion of resources away from reproductive performance towards somatic maintenance (the disposable soma interpretation [25]). What was unexpected was that this change was not also mirrored by a reduction in the size of the testes which were actually the third most protected organ. The relative protection of the testes under CR was also observed previously [28, 29]. This protection of the testes begs the question of whether reproductive performance really is compromised by CR [26]. In female mice it has been found that mice under CR had improved rates of fertility and reproductive performance [57]. It is uncertain whether the same also true of males?

The preferential use of the kidneys was also unexpected given that several previous studies have suggested that the kidneys are preserved under CR. For example, after 6 months of CR the kidneys were 8% smaller compared to *ad libitum* fed rats, with an overall mass difference of 16% [30]. McCay and colleagues found the kidneys decreased proportionately to body mass in rats under CR (i.e. β = 1) [27], while Lowry commented that rats under restriction “maintained younger kidneys” [58]. Weindruch et al. (1986) found that kidney mass was relatively protected compared with total mass loss (ie β < 1) [28]. One interpretation might be that the kidney size declined preferentially in our study because of the lower protein contents in the CR diets and hence a lower load on the organ. Although there was some reduction in the kidney size in the PR animals this was much less than in the CR mice, and the difference between the two was highly significant in the 30CR v 30PR and 40CR v 40PR comparisons (Table 6). This interpretation is therefore not supported. Several other studies have also found relative preservation in the sizes of the heart, liver and particularly the brain under CR [27, 28, 30]. It was unclear from the present study the extent to which changes in the vital organs are reversible once restriction ends. It may be that the hierarchy of utilization reflects in part the differential ability to regrow the organs once restriction ends.

The brain was the most resistant organ to mass loss with decreases only evident at the most severe level of restriction. Since we did not perform any cognitive tests it is uncertain whether the observed 4.4% mass loss at 40CR had any negative impacts on brain function. Among the more surprising observations was that the tail was the second most preserved organ after the brain. This is probably not related to its functional importance. Several rodent lineages have much reduced or even absent tails (e.g. voles and hamsters) and seem to survive well without them. Hence, it would not appear essential for survival. Its apparent preservation probably has more to do with the fact it consists mostly of bone, tendons and skin, and hence has relatively little utilizable energy in it that can be withdrawn and utilized.

Overall the reductions in the sizes of the vital organs provided very little energy towards the shortfall in intake (Table 8). Summed together they contributed about 4% of the total released energy (Table 8), and this total at 20-40% CR was about 18 % of the shortfall during the first month of restriction. Thus the reductions in the sizes of the vital organs contributed in total less than 1% to the energy shortfall. Why then do animals compromise the functions of these vital tissues under CR, when the benefit seems trivial? Why would a mouse risk reducing the size of its brain by 4.4 % when by doing so it only releases 0.12 kJ of energy? One possible reason is that such tissues are the principal sites where energy is utilized [24]. By reducing the sizes of these tissues the animal might dramatically reduce its energy needs thereby bringing its energy requirements back into line with the restricted energy supply. This explains why the changes in the sizes of these organs were linearly related to the extent of restriction, since the demand for reduced energy expenditure to balance intake is directly related to the restriction level. We will address elsewhere whether the changes in these organ sizes are sufficient to reduce energy demands to match supply (Mitchell et al., in prep).

One of the suggested potential downsides of CR is a negative impact on skeletal health [27, 59]. However, our results offer no evidence that three months of CR, even at the 40% level, had a negative effect on bone composition or mechanics. In fact one could conclude that there was a beneficial effect of CR on bone mass in the current study. The causes of these changes are uncertain. However, they may be correlated to hormonal and activity changes in the animals. The reduced fat mass under CR produced a reduction in levels of circulating leptin (Mitchell et al., in prep). Although contradictory reports exist [60, 61] leptin has been identified as a major inhibitor of bone mass accrual [62] with a higher bone mass phenotype observed in the leptin-deficient ob/ob mouse [63, 64]. Additionally, changes in the physical activity patterns of the mice under CR may have had an impact on bone structure. Mice on CR display extreme food anticipatory activity (FAA) behavior, a phenomenon related to their constant hunger [65, 66]. Weight-bearing physical activity plays an important role in bone health [67, 68] and the structure and composition of bone adapts to match the mechanical demands placed on it, which may have led to the longer and larger bones observed in the CR groups.

### Protein restriction

Most CR protocols, like ours, restrict the total diet and hence provide not only fewer calories but also proportionately lower levels of all the macronutrients, perhaps chief among which is the level of protein supply [2]. In fact it has been argued that the impact of CR may be due primarily to the reduced intake of protein, rather than reduced intake of calories [32, 69, 70]. We were interested, therefore, in the extent to which the observed changes in body composition under CR might be explained by changes in PR, and thus ran a second experiment in which protein was restricted without a calorie deficit to determine what aspects of the CR changes would be recapitulated under PR alone.

We observed that under PR the body composition remained virtually unchanged. Moreover, none of the observed differences in individual organ sizes after three months of PR reached statistical significance compared to the 12AL group. However, there was an overall significant impact on the total fat mass (summed across depots). As PR increased the animals became fatter over the three month manipulation period. Since the animals were provided with the same total calorie intake as during the baseline period and the same intake across the different PR groups, the most likely reason for this effect on fatness was that reducing the levels of protein in the diet reduced the specific dynamic action (SDA) of the diet, which is known to be greatest for the protein component [71]. Thus while gross energy intake remained constant the net metabolizable energy increased as the protein level declined. This would lead to surplus energy above requirements that the animals could deposit as fat. Since the extra fat in the 40PR group amounted to just over 1 g (39.5 kJ), this was equivalent to less than 0.3 kJ/day over the 90 day experiment and hence entirely consistent in magnitude with an alteration in the level of SDA.

The only organs that did not differ between the CR and PR treatments were those organs that were invested in or highly protected under CR (Figure 6 and Table 6). All the tissues that lost significant mass only did so under the CR and not under the PR treatments (compare Figures 5 and 10). Since the PR experiments recapitulated none of the major changes observed under equivalent levels of CR, we conclude that the major alterations in body composition of mice under CR come about entirely because of the restriction of calories, and the need to make good the immediate shortfall of energy and to match longer term energy demands to the diminished supply. The former is achieved primarily by withdrawing energy from the fat reserves and structural tissues (e.g. skin and skeletal muscle) and the latter by reducing the sizes of the vital organs.

## MATERIALS AND METHODS

### Overall design and rationale

We characterized the body composition response to CR and PR in C57BL/6 male mice, a strain known to have a positive lifespan response under CR [9]. The time-point at which CR is started has an impact on the lifespan effect. Initiation of CR at 4 weeks of age shortened lifespan [72] while CR introduced at 6 weeks increased lifespan [1]. Nevertheless both these early start points impact development, and are probably unrealistic models for implementation of CR in humans. Here mice were introduced to CR or PR at 20 weeks of age, approximately equivalent to early human adulthood, and close to the time when mice reach skeletal maturity [73]. This start time avoids impacts of CR on developmental processes. Previous studies suggested CR begun at six months was as effective at increasing lifespan as starting at 6 weeks [74].

A linear relationship between the extent of CR and the magnitude of the lifespan effect has also been indicated, up to at least a restriction of 65% which led to a 60% increase in lifespan [28, 65, 75]. We therefore exposed mice to 5 different levels of CR: 0, 10, 20, 30 and 40% lower calories than their own individual intakes measured over a baseline period of 14 days prior to introducing the restricted diets. Mice on restriction were individually housed and fed daily at lights out (1830h). There is a potential issue with an appropriate control group in CR studies [2, 76]. Animals that are fed completely *ad libitum* (AL) may become obese and hence the comparison of CR to AL animals may simply reflect an anti-obesity effect of CR. This is less of an issue when graded levels of CR are used instead of a single comparison of one CR level to AL animals. A further problem however occurs in relationship to terminal measurements. When animals are under CR they generally consume their food during the first few hours after it has been provided. They then have a protracted period without food before the next daily allocation of food arrives. AL animals in contrast can by definition eat at any time throughout the 24h period. Consequently, when it comes to culling animals to perform molecular biology work the CR animals may have been starving for 10-16 h, while the AL animals may have eaten in the hour immediately prior to culling. The CR v AL comparison may then be confounded by an immediate ‘time since last meal’ effect. To avoid these issues we used 2 ‘control groups’ exposed to 0% CR. For the first group (24AL) we allowed them 24h access to food without restriction. For the second group (12AL) we allowed them unrestricted access to food for the 12h of darkness but then removed the food at lights on (0630h), replacing it 12h later at lights off when the CR animals were also fed. Hence these animals, like the CR animals, had been starving for at least 7.5 h when we came to cull them between 1400 and 1800h.

All animals were fed a high carbohydrate open source diet (D12450B: Research diets, NJ, USA) which contains 20% protein, 70% carbohydrate and 10% fat (by energy). For the animals on PR we started with the same diet containing 20% protein as the control group. We then modified this diet by reducing its protein level and replacing the missing protein with carbohydrate to achieve protein levels of 16, 14 and 12 % protein. Animals on these protein diets were prevented from overeating to compensate for the reduced protein and were fed a fixed weight of food equivalent to their own individual baseline intake on the 20% protein diet. Hence their energy intakes were the same as during the baseline period but their protein intakes were restricted by 20, 30 and 40%, to match the protein levels consumed by the 20, 30 and 40% CR groups (D13020201, D13020202 and D13020203 respectively, Research Diets, NJ, USA). To match the CR protocol these animals were also only fed in darkness. For both studies the period of restriction was set at three months.

The overall aim of the study was to collect extensive phenotype data across the 7-9 animals in each group. These data included transcriptomic, proteomic and metabolomic profiles in multiple tissues, physiological, endocrinological and behavioral responses, as well as morphological changes. The focus of the current paper includes the changes in daily food intake, body mass, digestive efficiency, Dual-energy x-ray absorptiometry (DXA) measures throughout the restriction period, and primarily detailed aspects of the body composition changes established after the three months of restriction were complete. Future papers will address the other outcome measures.

### Animals

#### Ethics statement

All procedures were reviewed and approved by University of Aberdeen ethical approval committee and carried out under a Home Office issued license compliant with the Animals (Scientific Procedures) Act 1986.

C57BL/6 mice were purchased from Charles River (Ormiston, UK). Free access to water was provided. Body mass and food intake were recorded daily, immediately prior to feeding. Over a 2 week baseline period a number of measures were taken among which are reported here: DXA, and digestive efficiency measures. Mice were allocated into 6 experimental groups matched for body mass. Prior to culling all parameters measured at baseline were repeated and referred to as the final measures (F). Mice were killed approximately 4 hours prior to lights out from 1400 to 1800 h by a terminal CO_2_ overdose. After death a blood sample was collected by heart puncture. Brains were removed, weighed and frozen in isopentane over dry ice. All remaining tissues were rapidly removed (~10mins), weighed, divided appropriately for future analysis and snap frozen in liquid nitrogen. The liver was divided into 7 pieces and individually frozen in cryovials to avoid freeze/thaw artefacts. Any apparent disease states were recorded. The tibia and femur of the right leg were preserved by wrapping in phosphate-buffered saline (PBS) soaked tissue, sealed in plastic bags and stored at −20°C for analysis of mechanical properties. The tibia and femur of the left leg were fixed in 3.7% formaldehyde, and scanned by micro-computed tomography (micro-CT). For full details on methods please refer to [77, 78]. Precise measurements of length and diameter of both tibia and femur were recorded using a digital micrometer (± 0.01 mm) (RS 572-044, Mitutoyo, Andover, UK) and the mechanical properties were evaluated by three-point bending using an Instron 5564 testing machine (Instron, High Wycombe, UK). MathCAD software was used for analysis of data (Mathsoft Engineering and Education Inc., Cambridge, MA, USA). Ultrasound was used to measure the speed of sound in a bone slice using a pulser receiver (Model 5052 PR, Panametrics Inc, Waltham, MA, USA) and an oscilloscope (Hitachi V-665A, Tokyo, Japan). The density of the cortical bone was determined using Archimedes' principle. Finally, the water, organic and mineral contents of the bones were calculated from wet, dry (24h at 105ÐC) and ashed (24h at 600ÐC) weights. The left tibia and femur of 12AL control (*n* = 5) and 30CR mice (*n* = 4) were analyzed by three-dimensional micro-CT using Skyscan 1072 X-ray Microtomograph Scanner (Skyscan, Aartselaar, Belgium). Skyscan Nrecon software was used to reconstruct the images using a modified Feldkamp algorithm to obtain a three-dimension image which was then analyzed using the software CTAN. The fractional bone volume, (i.e. the percentage of bone volume relative to the total volume (BV/TV), trabecular thickness (Tb Th), trabecular separation (Tb Sp), trabecular number (Tb N), trabecular pattern factor (Tb Pf), the structural model index (SMI) and the degree of anisotropy (DoA) were recorded).

### Dual energy X-ray absorptiometry (DXA)

Fat mass, fat-free mass, bone mineral density (BMD), content (BMC) and bone area (BA) were quantified using DXA (GE PIXImus2 Series Densitometers installed with software version 1.46.007) (GE Medical Systems Ultrasound and BMD, UK) [79]. Measurements were taken at baseline, 4 and 8 weeks after restriction started and 3-4 days prior to the final kill.

### Bomb calorimetry

Feces collected over 6 days during baseline and following 11-12 weeks of restriction, were carefully separated from sawdust, weighed and dried along with a sample of each diet. Gross energy content for each diet or fecal sample was measured by bomb calorimetry (Parr 6100 calorimeter using a semi-micro 1109 oxygen bomb 1109A, Scientific and Medical Products Ltd, Cheadle, UK) with a minimum of three replicates, within ±0.25 kJ. Metabolizable energy intake (MEI) (kJ/day) was calculated from the gross energy intake (GEI) and energy output assuming a 3% energy loss via urine [80, 81]. The apparent energy absorption efficiency was calculated as the percentage of the ingested food taken up by the body.

### Statistical analysis

Statistical analyses were performed using the PASW Statistics package 18, Minitab version 16 and R. All data were first checked for normality using the Kolmogorov-Smirnov test and if necessary were normalized by log transformation prior to analysis. Unless otherwise stated general linear models (GLM), were used to compare data across time with individual ID entered into the model as a random factor nested within group to account for repeated measures (RM). Where time was not a factor (e.g. comparing groups at baseline) we used one way ANOVA. Where appropriate, following GLM or one way ANOVA post-hoc Tukey tests were used, with a significance threshold set at *p* < 0.05, to isolate differences between the 6 diet groups at specified time points. Pairwise comparisons were also occasionally made using *t*-tests when appropriate, for example when comparing the two AL fed groups, or paired *t*-tests when comparing animals to their own baseline measurements. We fitted second order polynomial relationships to the time courses of weight change over the initial period of exposure to the diets, and used the fitted coefficients to calculate the rates of initial change and the time to inflection in the mass loss curves. These latter parameters when analyzed in relation to the extent of restriction using non-linear regression analysis and one way ANOVA respectively.

For the detailed body composition analysis we probed the responses of individual tissues as a function of the level of restriction using GLM with initial body mass as a covariate in the analysis, followed where appropriate by post-hoc Tukey tests. We performed a Pearson correlation analysis of the responses of different tissues, and analyzed the patterns of response across the different tissues using clustering analysis, with complete linkage and the distances based on correlation coefficients. This suggested common patterns among the vital organs, structural components, adipose tissue and the alimentary tract components and hence we pooled the individual measures into these functional groups as well, before evaluating the impact of CR on these functional groupings using GLM. To evaluate the relative importance of tissue level changes compared with the overall weight loss we plotted individual tissue weights against the final body mass at the point of death and fitted linear (least squares) relationships to these functions. The gradients (β) of these relationships provided a measure of whether a particular tissue was disproportionately withdrawn (β > 1.0), relatively protected (β ≤ 1.0 and ≥ 0.0) or invested in (β < 0.0). Finally we used literature values of the tissue energy contents to assess the contribution of different tissue withdrawal to the total energy shortfall during restriction.

## SUPPLEMENTARY MATERIALS TABLES



## References

[1] Weindruch R, Walford RL (1982). Dietary restriction in mice beginning at 1 year of age: Effect on life-span and spontaneous cancer incidence. Science.

[2] Speakman JR, Mitchell SE (2011). Caloric restriction. Mol Aspects Med.

[3] Osborne TB, Mendel LB, Ferry EL (1917). The effect of retardation of growth upon the breeding period and duration of life in rats. Science.

[4] Weindruch R, Walford R.L. (1988). The retardation of aging and disease by dietary restriction.

[5] Colman RJ, Beasley TM, Allison DB, Weindruch R (2008). Attenuation of sarcopenia by dietary restriction in rhesus monkeys. The Journals of Gerontology Series A: Biological Sciences and Medical Sciences.

[6] Merry BJ (2002). Molecular mechanisms linking calorie restriction and longevity. Int J Biochem Cell Biol.

[7] Speakman JR, Hambly C (2007). Starving for life: What animal studies can and cannot tell us about the use of caloric restriction to prolong human lifespan. J Nutr.

[8] Rizza W, Veronese N, Fontana L (2014). What are the roles of calorie restriction and diet quality in promoting healthy longevity?. Ageing Research Reviews.

[9] Turturro A, Witt WW, Lewis S, Hass BS, Lipman RD, Hart RW (1999). Growth curves and survival characteristics of the animals used in the biomarkers of aging program. The Journals of Gerontology Series A: Biological Sciences and Medical Sciences.

[10] Forster M, Morris P, Sohal R (2003). Genotype of age influence the effect of caloric intake on mortality in mice. FASEB J.

[11] Sohal RS, Ferguson M, Sohal BH, Forster MJ (2009). Life span extension in mice by food restriction depends on an energy imbalance. J Nutr.

[12] Liao C, Rikke BA, Johnson TE, Diaz V, Nelson JF (2010). Genetic variation in the murine lifespan response to dietary restriction: From life extension to life shortening. Aging Cell.

[13] Hempenstall S, Picchio L, Mitchell SE, Speakman JR, Selman C (2010). The impact of acute caloric restriction on the metabolic phenotype in male C57BL/6 and DBA/2 mice. Mech Ageing Dev.

[14] Mulvey L, Sinclair A, Selman C (2014). Lifespan modulation in mice and the confounding effects of genetic background. Journal of Genetics and Genomics.

[15] Masoro EJ (1996). Possible mechanisms underlying the antiaging actions of caloric restriction. Toxicol Pathol.

[16] Millet L, Vidal H, Andreelli F, Larrouy D, Riou JP, Ricquier D, Laville M, Langin D (1997). Increased uncoupling protein-2 and -3 mRNA expression during fasting in obese and lean humans. J Clin Invest.

[17] Picard F, Kurtev M, Chung N, Topark-Ngarm A, Senawong T, De Oliveira RM, Leid M, McBurney MW, Guarente L (2004). Sirt1 promotes fat mobilization in white adipocytes by repressing PPAR-γ. Nature.

[18] Bordone L, Guarente L (2005). Calorie restriction, SIRT1 and metabolism: Understanding longevity. Nat Rev Mol Cell Biol.

[19] Colom B, Oliver J, Roca P, Garcia-Palmer FJ (2007). Caloric restriction and gender modulate cardiac muscle mitochondrial H2O2 production and oxidative damage. Cardiovasc Res.

[20] Barzilai N, Bartke A (2009). Biological approaches to mechanistically understand the healthy life span extension achieved by calorie restriction and modulation of hormones. The Journals of Gerontology Series A: Biological Sciences and Medical Sciences.

[21] Zhang Y, Proenca R, Maffei M, Barone M, Leopold L, Friedman JM (1994). Positional cloning of the mouse obese gene and its human homologue. Nature.

[22] Ahima RS, Flier JS (2000). Adipose tissue as an endocrine organ. Trends in Endocrinology and Metabolism.

[23] Hambly C, Speakman JR (2005). Contribution of different mechanisms to compensation for energy restriction in the mouse. Obes Res.

[24] Elia M (1992). Organ and tissue contribution to metabolic rate. Energy Metabolism: Tissue Determinants and Cellular Corollaries.

[25] Kirkwood TB, Holliday R (1979). The evolution of ageing and longevity. Proc R Soc Lond B Biol Sci.

[26] Flatt T (2011). Survival costs of reproduction in drosophila. Exp Gerontol.

[27] McCay CM, Crowell MF, Maynard LA (1935). The effect of retarded growth upon the length of life span and upon the ultimate body size. J Nutr.

[28] Weindruch R, Walford RL, Fligiel S, Guthrie D (1986). The retardation of aging in mice by dietary restriction: Longevity, cancer, immunity and lifetime energy intake. J Nutr.

[29] Weindruch R, Sohal RS (1997). Caloric intake and aging. N Engl J Med.

[30] Selman C, Phillips T, Staib JL, Duncan JS, Leeuwenburgh C, Speakman JR (2005). Energy expenditure of calorically restricted rats is higher than predicted from their altered body composition. Mech Ageing Dev.

[31] Cameron KM, Golightly A, Miwa S, Speakman J, Boys R, von Zglinicki T (2011). Gross energy metabolism in mice under late onset, short term caloric restriction. Mech Ageing Dev.

[32] Simpson SJ, Raubenheimer D (2009). Macronutrient balance and lifespan. Aging.

[33] Speakman JR (2005). Body size, energy metabolism and lifespan. Journal of Experimental Biology.

[34] Krol E, Speakman JR (1999). Isotope dilution spaces of mice injected simultaneously with deuterium, tritium and oxygen-18. J Exp Biol.

[35] Hall KD, Jordan PN (2008). Modeling weight-loss maintenance to help prevent body weight regain. Am J Clin Nutr.

[36] Gelegen C, Collier DA, Campbell IC, Oppelaar H, Kas MJ (2006). Behavioral, physiological, and molecular differences in response to dietary restriction in three inbred mouse strains. Am J Physiol Endocrinol Metab.

[37] Lee M, Lucia S (1961). Some relationships between caloric restriction and body weight in the rat I. body composition, liver lipids and organ weights. J Nutr.

[38] Yang H, Youm Y, Nakata C, Dixit VD (2007). Chronic caloric restriction induces forestomach hypertrophy with enhanced ghrelin levels during aging. Peptides.

[39] Merry B, Goldspink D, Lewis S (1991). The effects of age and chronic restricted feeding on protein synthesis and growth of the large intestine of the rat. Comparative Biochemistry and Physiology Part A: Physiology.

[40] Merry BJ, Lewis SE, Goldspink DF (1992). The influence of age and chronic restricted feeding on protein synthesis in the small intestine of the rat. Exp Gerontol.

[41] Casirola DM, Rifkin B, Tsai W, Ferraris RP (1996). Adaptations of intestinal nutrient transport to chronic caloric restriction in mice. American Journal of Physiology - Gastrointestinal and Liver Physiology.

[42] Casirola DM, Lan Y, Ferraris RP (1997). Effects of changes in calorie intake on intestinal nutrient uptake and transporter mRNA levels in aged mice. The Journals of Gerontology Series A: Biological Sciences and Medical Sciences.

[43] Bertrand HA, Lynd FT, Masoro EJ, Yu BP (1980). Changes in adipose mass and cellularity through the adult life of rats fed ad libitum or a life-prolonging restricted diet. J Gerontol.

[44] Bertrand HA, Stacy C, Masoro EJ, Yu BP, Murata I, Maeda H (1984). Plasticity of fat cell number. J Nutr.

[45] Bagga D, Byerley LO, Koziol BJ, Glick Z, Ashley JM, Heber D (1995). Adipose tissue and the effects of fat and calories on breast tumorigenesis in rats. J Nutr Biochem.

[46] Barzilai N, Banerjee S, Hawkins M, Chen W, Rossetti L (1998). Caloric restriction reverses hepatic insulin resistance in aging rats by decreasing visceral fat. J Clin Invest.

[47] Das SK, Gilhooly CH, Golden JK, Pittas AG, Fuss PJ, Cheatham RA, Tyler S, Tsay M, McCrory MA, Lichtenstein AH, Dallal GE, Dutta C, Bhapkar MV (2007). Long-term effects of 2 energy-restricted diets differing in glycemic load on dietary adherence, body composition, and metabolism in CALERIE: A 1-y randomized controlled trial. The American Journal of Clinical Nutrition.

[48] Redman LM, Heilbronn LK, Martin CK, Alfonso A, Smith SR, Ravussin E, Pennington CALERIE Team (2007). Effect of calorie restriction with or without exercise on body composition and fat distribution. J Clin Endocrinol Metab.

[49] Nicklas BJ, Wang X, You T, Lyles MF, Demons J, Easter L, Berry MJ, Lenchik L, Carr JJ (2009). Effect of exercise intensity on abdominal fat loss during calorie restriction in overweight and obese postmenopausal women: A randomized, controlled trial. Am J Clin Nutr.

[50] Merry B (2002). Molecular mechanisms linking calorie restriction and longevity. Int J Biochem Cell Biol.

[51] Barzilai N, Gupta G (1999). Interaction between aging and syndrome X: New insights on the pathophysiology of fat distribution. Ann N Y Acad Sci.

[52] Picard F, Guarente L (2005). Molecular links between aging and adipose tissue. Int J Obes.

[53] Muzumdar R, Allison DB, Huffman DM, Ma X, Atzmon G, Einstein FH, Fishman S, Poduval AD, McVei T, Keith SW, Barzilai N (2008). Visceral adipose tissue modulates mammalian longevity. Aging Cell.

[54] Harrison DE, Archer JR, Astle CM (1984). Effects of food restriction on aging: Separation of food intake and adiposity. Proc Natl Acad Sci U S A.

[55] Liao C, Rikke BA, Johnson TE, Gelfond JAL, Diaz V, Nelson JF (2011). Fat maintenance is a predictor of the murine lifespan response to dietary restriction. Aging Cell.

[56] Stiegler P, Cunliffe A (2006). The role of diet and exercise for the maintenance of fat-free mass and resting metabolic rate during weight loss. Sports Medicine.

[57] Johnston SL, Grune T, Bell LM, Murray SJ, Souter DM, Erwin SS, Yearsley JM, Gordon IJ, Illius AW, Kyriazakis I, Speakman JR (2006). Having it all: Historical energy intakes do not generate the anticipated trade-offs in fecundity. Proc Biol Sci.

[58] Lowry OH, Hastings AB (1942). Histochemical changes associated with aging I. methods and calculations. J Biol Chem.

[59] Baek K, Barlow AA, Allen MR, Bloomfield SA (2008). Food restriction and simulated microgravity: Effects on bone and serum leptin. Journal of Applied Physiology.

[60] Gat-Yablonski G, Ben-Ari T, Shtaif B, Potievsky O, Moran O, Eshet R, Maor G, Segev Y, Phillip M (2004). Leptin reverses the inhibitory effect of caloric restriction on longitudinal growth. Endocrinology.

[61] Hamrick MW, Pennington C, Newton D, Xie D, Isales C (2004). Leptin deficiency produces contrasting phenotypes in bones of the limb and spine. Bone.

[62] Ducy P, Kousteni S (2015). Leptin and bone. In Leptin.

[63] Ducy P, Amling M, Takeda S, Priemel M, Schilling AF, Beil FT, Shen J, Vinson C, Rueger JM, Karsenty G (2000). Leptin inhibits bone formation through a hypothalamic relay: A central control of bone mass. Cell.

[64] Karsenty G (2001). Leptin controls bone formation through a hypothalamic relay. Recent Progress in Hormone Research.

[65] Hambly C, Mercer JG, Speakman JR (2007). Hunger does not diminish over time in mice under protracted caloric restriction. Rejuvenation Res.

[66] Hambly C, Duncan JS, Archer ZA, Moar KM, Mercer JG, Speakman JR (2012). Repletion of TNFα or leptin in calorically restricted mice suppresses post-restriction hyperphagia. Disease Models & Mechanisms.

[67] Macdonald HM, Ashe MC, McKay HA (2009). The link between physical activity and bone strength across the lifespan. International Journal of Clinical Rheumatology.

[68] Wallace IJ, Tommasini SM, Judex S, Garland T, Demes B (2012). Genetic variations and physical activity as determinants of limb bone morphology: An experimental approach using a mouse model. Am J Phys Anthropol.

[69] Mair W, Piper MD, Partridge L (2005). Calories do not explain extension of life span by dietary restriction in drosophila. PLoS Biology.

[70] Solon-Biet SM, McMahon AC, Ballard JWO, Ruohonen K, Wu LE, Cogger VC, Warren A, Huang X, Pichaud N, Melvin RG (2014). The ratio of macronutrients, not caloric intake, dictates cardiometabolic health, aging, and longevity in ad libitum-fed mice. Cell Metabolism.

[71] Borsook H (1936). The specific dynamic action of protein and amino acids in animals. Biological Reviews.

[72] Harrison DE, Archer JR (1987). Genetic differences in effects of food restriction on aging in mice. J Nutr.

[73] Somerville J, Aspden R, Armour K, Armour K, Reid D (2004). Growth of C57BL/6 mice and the material and mechanical properties of cortical bone from the tibia. Calcif Tissue Int.

[74] Yu BP, Masoro EJ, McMahan CA (1985). Nutritional influences on aging of fischer 344 rats: I. physical, metabolic, and longevity characteristics. Journal of Gerontology.

[75] Merry B (2002). Molecular mechanisms linking calorie restriction and longevity. Int J Biochem Cell Biol.

[76] Sohal RS, Forster MJ (2014). Caloric restriction and the aging process: A critique. Free Radical Biology and Medicine.

[77] Aspden R (2003). Mechanical testing of bone ex vivo. In Bone Research Protocols.

[78] Goodyear S, Aspden R (2012). Mechanical properties of bone ex vivo. In Bone Research Protocols.

[79] Johnston SL, Peacock WL, Bell LM, Lonchampt M, Speakman JR (2005). PIXImus DXA with different software needs individual calibration to accurately predict fat mass. Obesity.

[80] Drozdz A (1975). Metabolic cages for small rodents. Methods for Ecological Bioenergetics.

[81] Krol E, Speakman JR (2003). Limits to sustained energy intake. VI. energetics of lactation in laboratory mice at thermoneutrality. J Exp Biol.

