# Supplementary material for: The effects of graded levels of calorie restriction: I. impact of short term calorie and protein restriction on body composition in the C57BL/6 mouse
Source: Oncotarget. 2015 Jun 6;6(18):15902–30. doi: 10.18632/oncotarget.4142 (PMC4599246; doi:10.18632/oncotarget.4142)
Supplement: Supplementary file 1 [file oncotarget-06-15902-s001.pdf]

The effects of graded levels of calorie restriction: I. impact of short term calorie and protein restriction on body composition in the C57BL/6 mouse

Supplementary Material

Supplementary Table 1

Fitted second order polynomial equations to the weight loss trajectories observed over the first 30 days of calorie restriction (CR). Curves were fitted to the equation $y = a.x^2 + b.x + c$ and the r^2 and coefficients of the fits are shown with the calculated inflection point. Treatment is the level of CR (10, 20, 30 and 40%) and ID is the individual ID number of the animal in the study. For one animal there was no fit ($r^2 < .15$).

Treatment	ID	r^2	a	b	C	inflection (days)
10CR	8	0.4078	0.00279	-0.08860	25.176	15.9
	9	0.908	0.00957	-0.35308	26.840	18.4
	21	0.5418	0.00078	-0.04039	28.602	25.9
	33	0.7248	0.00029	-0.03579	28.477	61.7
	46	0.3469	0.00159	-0.06742	27.221	21.2
	50	0.8744	0.00293	-0.15619	30.757	26.7
	54	0.9245	0.00253	-0.13168	28.335	26.1
	56	0.8641	0.00404	-0.16629	26.508	20.6
20CR	39	No fit				
	4	0.6351	0.00119	-0.06823	27.475	28.6
	10	0.8499	0.00232	-0.12314	26.336	26.5
	27	0.9184	0.00338	-0.21498	27.870	31.8
	37	0.8386	0.00556	-0.23642	30.540	21.3
	47	0.9571	0.00621	-0.27505	31.337	22.2
	57	0.9811	0.00397	-0.25876	26.790	32.6
	64	0.9737	0.00675	-0.33271	28.830	24.6
30CR	6	0.9705	0.00994	-0.46422	28.567	23.3
	24	0.9405	0.00553	-0.27052	28.227	24.5
	36	0.9726	0.00350	-0.23060	28.365	32.9
	49	0.9866	0.00960	-0.46470	26.716	24.2
	52	0.9740	0.00890	-0.56930	31.060	32.0
	53	0.9932	0.00260	-0.31140	31.810	59.9
	55	0.9904	0.00850	-0.48610	29.264	28.6
40CR	7	0.9815	0.01140	-0.51800	27.849	22.7
	28	0.9915	0.01220	-0.58850	28.149	24.1
	30	0.9910	0.01010	-0.50850	26.547	25.2
	34	0.9937	0.00660	-0.51030	29.058	38.7
	44	0.9524	0.00340	-0.42140	30.399	62.0
	48	0.9956	0.00800	-0.51660	28.082	32.3

58	0.9936	0.01160	-0.52800	25.445	22.8
62	0.9830	0.00920	-0.52960	28.294	28.8
67	0.9919	0.01500	-0.71120	28.888	23.7

Supplementary Table 2

Pearson correlation coefficients and associated p values relating the masses of different organs across individuals.

A: Caloric restriction

	**Log brain**	**Log liver**	**Log kidneys**	**Log heart**
Log liver	0.268			
	0.066			
Log kidneys	0.745	0.495		
	<0.0001	<0.0001		
Log heart	0.547	0.386	0.731	
	<0.0001	0.007	<0.0001	
Log spleen	0.717	0.436	0.725	0.644
	<0.0001	0.002	<0.0001	<0.0001
Log lungs	0.498	0.350	0.502	0.382
	<0.0001	0.015	<0.0001	0.007
Log pancreas	0.412	0.286	0.663	0.524
	0.004	0.049	<0.0001	<0.0001
Log EPI	0.679	0.551	0.715	0.608
	<0.0001	<0.0001	<0.0001	<0.0001
Log retro	0.661	0.680	0.798	0.622
	<0.0001	<0.0001	<0.0001	<0.0001
Log omental	0.446	0.374	0.533	0.460
	0.001	0.009	<0.0001	0.001
Log sub cut	0.680	0.639	0.768	0.640
	<0.0001	<0.0001	<0.0001	<0.0001
Log BAT	0.625	0.624	0.747	0.560
	<0.0001	<0.0001	<0.0001	<0.0001
BC testes	0.372	0.345	0.323	0.336
	0.009	0.016	0.025	0.019
Log rep orgs	0.341	0.764	0.524	0.361
	0.018	<0.0001	<0.0001	0.012
Log tail	0.316	0.533	0.326	0.179
	0.029	<0.0001	0.024	0.222
Log carcass	0.724	0.673	0.848	0.703
	<0.0001	<0.0001	<0.0001	<0.0001
Log skin	0.613	0.721	0.787	0.661
	<0.0001	<0.0001	<0.0001	<0.0001
Log stomach	-0.195	-0.396	-0.445	-0.348
	0.185	0.005	0.002	0.015
Log ileum	-0.048	-0.031	-0.180	0.038
	0.744	0.835	0.220	0.799
BC caecum	0.057	0.124	-0.012	-0.070
	0.701	0.401	0.936	0.637
Log colon	-0.159	-0.204	-0.080	0.088
	0.281	0.164	0.587	0.551

	Log spleen	Log lungs	Log pancreas	Log EPI
Log lungs	0.383			
	0.007			
Log pancreas	0.455	0.272		
	0.001	0.062		
Log EPI	0.704	0.419	0.409	
	<0.0001	0.003	0.004	
Log retro	0.685	0.432	0.495	0.853
	<0.0001	0.002	<0.0001	<0.0001
Log omental	0.632	0.373	0.244	0.393
	<0.0001	0.009	0.095	0.006
Log sub cut	0.701	0.458	0.445	0.909
	<0.0001	0.001	0.002	<0.0001
Log BAT	0.622	0.508	0.504	0.747
	<0.0001	<0.0001	<0.0001	<0.0001
BC testes	0.494	0.289	0.271	0.556
	<0.0001	0.046	0.063	<0.0001
Log rep orgs	0.590	0.203	0.255	0.609
	<0.0001	0.167	0.080	<0.0001
Log tail	0.290	0.322	0.198	0.270
	0.046	0.026	0.177	0.063
Log carcass	0.819	0.462	0.569	0.839
	<0.0001	0.001	<0.0001	<0.0001
Log skin	0.655	0.493	0.474	0.833
	<0.0001	<0.0001	0.001	<0.0001
Log stomach	-0.238	-0.090	-0.178	-0.434
	0.103	0.542	0.226	0.002
Log ileum	-0.059	0.033	-0.108	-0.209
	0.690	0.823	0.467	0.153
BC caecum	-0.034	0.323	0.017	-0.157
	0.821	0.025	0.908	0.286
Log colon	-0.109	-0.056	0.131	-0.222
	0.461	0.705	0.374	0.129

	Log retro	Log omental	Log subcut	Log BAT
Log omental	0.473			
	0.001			
Log sub cut	0.930	0.434		
	<0.0001	<0.0001		
Log BAT	0.830	0.493	0.834	
	<0.0001	<0.0001	<0.0001	
BC testes	0.494	0.211	0.503	0.346
	<0.0001	0.150	<0.0001	0.016
Log rep orgs	0.747	0.458	0.644	0.582
	<0.0001	0.001	<0.0001	<0.0001
Log tail	0.334	0.280	0.292	0.386
	0.020	0.054	0.044	0.007
Log carcass	0.916	0.593	0.892	0.823
	<0.0001	<0.0001	<0.0001	<0.0001

Log skin	0.871	0.485	0.878	0.809
	<0.0001	<0.0001	<0.0001	<0.0001
Log stomach	-0.475	-0.028	-0.449	-0.355
	0.001	0.852	0.001	0.013
Log ileum	-0.194	-0.023	-0.180	-0.129
	0.186	0.875	0.221	0.383
BC caecum	-0.003	0.207	-0.069	0.146
	0.985	0.158	0.643	0.324
Log colon	-0.242	-0.010	-0.299	-0.273
	0.098	0.947	0.117	0.060

	**Log testes**	**Log rep orgs**	**Log tail**	**Log carcass**
Log rep orgs	0.476			
	0.001			
Log tail	0.282	0.470		
	0.052	0.001		
Log carcass	0.525	0.755	0.422	
	<0.0001	<0.0001	0.003	
Log skin	0.436	0.718	0.481	0.907
	0.002	<0.0001	0.001	<0.0001
Log stomach	-0.093	-0.472	-0.077	-0.434
	0.529	0.001	0.601	0.002
Log ileum	-0.038	-0.181	-0.235	-0.1911
	0.798	0.219	0.108	0.193
BC caecum	0.044	-0.060	0.226	-0.041
	0.766	0.685	0.122	0.782
Log colon	-0.195	-0.367	-0.357	-0.226
	0.183	0.010	0.013	0.122

	**Log skin**	**Log stomach**	**Log ileum**	**Log caecum**
Log stomach	-0.446			
	0.001			
Log ileum	-0.216	0.219		
	0.141	0.134		
BC caecum	0.013	0.326	0.255	
	0.930	0.024	0.080	
Log colon	-0.310	0.362	0.278	-0.223
	0.032	0.011	0.055	0.128

B: Protein restriction

	**Brain**	**Liver**	**Heart**	**Kidneys**
Liver	-0.464			
	0.008			
Heart	0.484	-0.158		
	0.005	0.386		
Kidneys	0.456	-0.614	0.220	
	0.009	<0.0001	0.227	
Lungs	0.064	-0.126	-0.303	0.344
	0.728	0.492	0.092	0.054
Spleen	0.422	-0.625	0.204	0.785
	0.016	<0.0001	0.263	<0.0001
Pancreas	0.044	-0.395	0.229	0.399
	0.813	0.025	0.207	0.024
Retro	-0.287	0.704	-0.130	-0.308
	0.111	<0.0001	0.478	0.086
EPI	-0.155	0.579	-0.118	-0.040
	0.397	0.001	0.520	0.828
Sub but	-0.421	0.661	-0.207	-0.246
	0.018	<0.0001	0.264	0.182
BAT	-0.089	-0.029	0.114	0.478
	0.630	0.876	0.534	0.006
Mesenteric	0.025	0.107	-0.039	0.201
	0.892	0.560	0.831	0.270
Carcass	-0.193	0.195	0.212	-0.094
	0.290	0.285	0.244	0.607
Skin	-0.069	0.647	0.232	-0.204
	0.706	<0.0001	0.201	0.262
Tail	0.054	0.317	0.032	-0.062
	0.768	0.078	0.862	0.736
Rep orgs	-0.403	0.609	-0.181	-0.567
	0.022	<0.0001	0.320	0.001
Testes	-0.066	0.435	-0.093	-0.119
	0.718	0.013	0.613	0.516
Stomach	-0.005	0.179	0.026	0.021
	0.977	0.327	0.888	0.909
Colon	0.069	-0.150	0.075	0.145
	0.709	0.413	0.682	0.427
Ileum	0.159	0.010	0.227	-0.038
	0.386	0.957	0.211	0.836
Caecum	-0.094	0.071	-0.028	-0.378
	0.607	0.700	0.880	0.033

	**Lungs**	**Spleen**	**Pancreas**	**Retro**
Spleen	0.332			
	0.063			
Pancreas	0.066	0.240		
	0.718	0.186		

Retro	-0.110	-0.310	-0.361	
	0.550	0.084	0.042	
EPI	-0.023	-0.202	-0.338	0.862
	0.900	0.267	0.058	<0.0001
Sub cut	-0.083	-0.345	-0.172	0.795
	0.656	0.057	0.355	<0.0001
BAT	0.081	0.320	0.321	0.126
	0.692	0.074	0.074	0.490
Mesenteric	0.073	0.324	-0.367	0.435
	0.692	0.070	0.039	0.013
Carcass	0.025	-0.039	0.478	0.089
	0.753	0.834	0.006	0.629
Skin	-0.156	-0.272	-0.248	0.578
	0.395	0.132	0.170	0.001
Tail	0.267	0.021	-0.067	0.274
	0.140	0.908	0.714	0.130
Rep orgs	-0.024	-0.615	-0.403	0.434
	0.895	<0.0001	0.022	0.013
Testes	-0.002	0.110	-0.195	0.401
	0.993	0.549	0.285	0.023
Stomach	0.152	-0.087	0.040	-0.184
	0.405	0.636	0.830	0.313
Colon	-0.148	0.087	-0.036	-0.286
	0.418	0.636	0.846	0.112
Ileum	0.073	0.049	0.033	-0.248
	0.691	0.860	0.171	0.334
Caecum	-0.210	-0.211	-0.223	-0.048
	0.249	0.245	0.219	0.794

	**EPI**	**Sub cut**	**BAT**	**Mesenteric**
Sub cut	0.724			
	<0.0001			
BAT	0.201	0.265		
	0.271	0.149		
Mesenteric	0.499	0.235	0.218	
	0.004	0.204	0.231	
Carcass	-0.056	0.304	0.397	-0.242
	0.760	0.096	0.025	0.183
Skin	0.602	0.629	0.024	0.131
	<0.0001	<0.0001	0.897	0.474
Tail	0.186	0.231	-0.328	-0.008
	0.308	0.211	0.066	0.963
Rep orgs	0.428	0.485	-0.133	0.006
	0.014	0.006	0.469	0.976
Testes	0.293	0.286	0.038	0.229
	0.103	0.119	0.835	0.207
Stomach	-0.116	-0.031	0.159	-0.169
	0.527	0.870	0.386	0.355
Colon	-0.0006	-0.262	0.004	0.057

	0.972	0.154	0.981	0.755
Ileum	-0.176	-0.178	0.157	-0.241
	0.334	0.337	0.391	0.184
Caecum	-0.033	-0.158	-0.244	-0.076
	0.858	0.396	0.178	0.678

	**Carcass**	**Skin**	**Tail**	**Rep orgs**
Skin	0.192			
	0.293			
Tail	0.047	0.298		
	0.798	0.098		
Rep orgs	-0.003	0.413	0.195	
	0.987	0.019	0.285	
Testes	0.072	0.315	0.180	0.067
	0.695	0.079	0.325	0.716
Stomach	0.151	0.085	0.192	0.195
	0.410	0.645	0.293	0.285
Colon	-0.190	-0.061	-0.131	0.051
	0.297	0.740	0.474	0.782
Ileum	0.099	0.028	0.098	0.021
	0.591	0.881	0.594	0.908
Caecum	0.117	0.029	-0.089	0.182
	0.523	0.876	0.627	0.318

	**Testes**	**Stomach**	**Colon**	**Ileum**
Stomach	0.025			
	0.890			
Colon	-0.059	0.144		
	0.750	0.433		
Ileum	-0.076	0.356	0.122	
	0.679	0.046	0.507	
Caecum	-0.102	0.197	0.400	0.220
	0.579	0.281	0.023	0.227
